# Starvation decreases immunity and immune regulatory factor NF-κB in the starlet sea anemone *Nematostella vectensis*

**DOI:** 10.1038/s42003-023-05084-7

**Published:** 2023-07-07

**Authors:** Pablo J. Aguirre Carrión, Niharika Desai, Joseph J. Brennan, James E. Fifer, Trevor Siggers, Sarah W. Davies, Thomas D. Gilmore

**Affiliations:** 1 Department of Biology, Boston University, Boston, MA 02215 USA; 2 Present Address: Pfizer, Inc., 1 Portland St, Cambridge, MA 02139 USA

**Keywords:** Innate immunity, Molecular evolution

## Abstract

Lack of proper nutrition has important consequences for the physiology of all organisms, and nutritional status can affect immunity, based on many studies in terrestrial animals. Here we show a positive correlation between nutrition and immunity in the sea anemone *Nematostella vectensis*. Gene expression profiling of adult anemones shows downregulation of genes involved in nutrient metabolism, cellular respiration, and immunity in starved animals. Starved adult anemones also have reduced protein levels and activity of immunity transcription factor NF-κB. Starved juvenile anemones have increased sensitivity to bacterial infection and also have lower NF-κB protein levels, as compared to fed controls. Weighted Gene Correlation Network Analysis (WGCNA) is used to identify significantly correlated gene networks that were downregulated with starvation. These experiments demonstrate a correlation between nutrition and immunity in an early diverged marine metazoan, and the results have implications for the survival of marine organisms as they encounter changing environments.

## Abstract (web summary)

Starvation is found to decrease immunity via effects on NF-κB in the Starlet Sea Anemone <i>Nematostella vectensis<i>, advancing our understanding of the correlation between nutrition and immunity in marine metazoans.

## Introduction

Maintenance of caloric needs is a requirement across the tree of life. Food scarcity is a challenge most heterotrophs encounter at various points during their lives—a situation that contributes to natural selection. The lack of proper nutrition (malnutrition) or the complete absence of food (starvation) has important consequences for an organism’s physiology. Starvation can lead to the slowing of metabolism as a means to conserve energy^1^. Furthermore, nutrition is one of the many factors that determine immune status of organisms^2^. Indeed, the adverse impact of poor nutrition on the immune system, including its inflammatory component, is well documented in vertebrates and some terrestrial invertebrates^3–11^. However, the interplay between nutrition and immunity has, for the most part, only been described in organisms from insects to mammals, and is  understudied in early diverging lineages of invertebrates, such as the phylum Cnidaria.

The sea anemone *Nematostella vectensis* (*Nv*) is a cnidarian that shows remarkable adaptability—being able to survive across a wide range of salinity, pH, and temperature—and it can be readily maintained and studied in the laboratory^12^. *Nv* also has exceptional regenerative abilities, being able to replace its entire oral end in ~6 days following bisection^13,14^. Importantly, *Nv* can withstand periods of starvation for over a month, where its body size decreases in response to the lack of caloric intake while maintaining body proportionality^15^, suggesting that *Nv* can physiologically respond to changes in food availability. The ability to respond to food availability is also seen in young *Nv*, as the timing of tentacle development in juvenile *Nv* polyps is affected by feeding regimens^16^. *Nv* is also distantly related to reef-building corals, where increased food availability has been shown to mitigate negative consequences of warming waters^17–19^. Thus, multiple cnidarians appear to have the ability to adjust physiological status based on nutritional intake and/or availability.

Among energetic needs, the immune system is metabolically costly and subject to complex regulation^20^. However, the relationship between nutrition and immunity in *Nv*—or any cnidarian—remains largely unexplored. Transcription factor NF-κB (nuclear factor kappa B) has been intensively studied in animals from insects to vertebrates for its involvement in immunity^21^. Examples of NF-κB target genes include cytokines and other immune response factors in mammals and anti-microbial proteins such as cecropins in insects^22,23^. Furthermore, NF-κB has been shown to play a role in metabolic disease in mammals, based on its role in inflammation and the association of chronic inflammation with diseases such as obesity and diabetes^6^.

Many “higher” metazoans (e.g., mammals and insects) have multiple NF-κB proteins, many of which are involved in some aspect of immunity^21^; however, most non-bilaterian animals, including sponges^24^ and cnidarians^25^, have single NF-κB proteins, and the biological roles of NF-κB in these organisms are less clear. In *Nv*, the NF-κB pathway has been linked to development^26^ and immunity^27,28^, and there is evidence that NF-κB activity is regulated at the transcriptional level in *Nv*^28^, as well as in several other early diverged metazoans^24,29,30^.

Herein, we have investigated the relationship between nutrition and immunity in *Nv* by 1) characterizing global gene expression changes in response to starvation, 2) assessing susceptibility to infection with a bacterial pathogen after starvation, 3) characterizing NF-κB after starvation by Western blotting, DNA-binding activity, and immunostaining, and 4) using Weighted Gene Correlation Network Analysis to explore relevant gene–gene interactions. Our results directly connect nutrition and immunity in a cnidarian, supporting the concept that nutritional status and immunity are linked processes across a broad evolutionary expanse.

## Results

### Genome-wide transcriptomic changes in *N. vectensis* in response to starvation

**Fig. 1:**
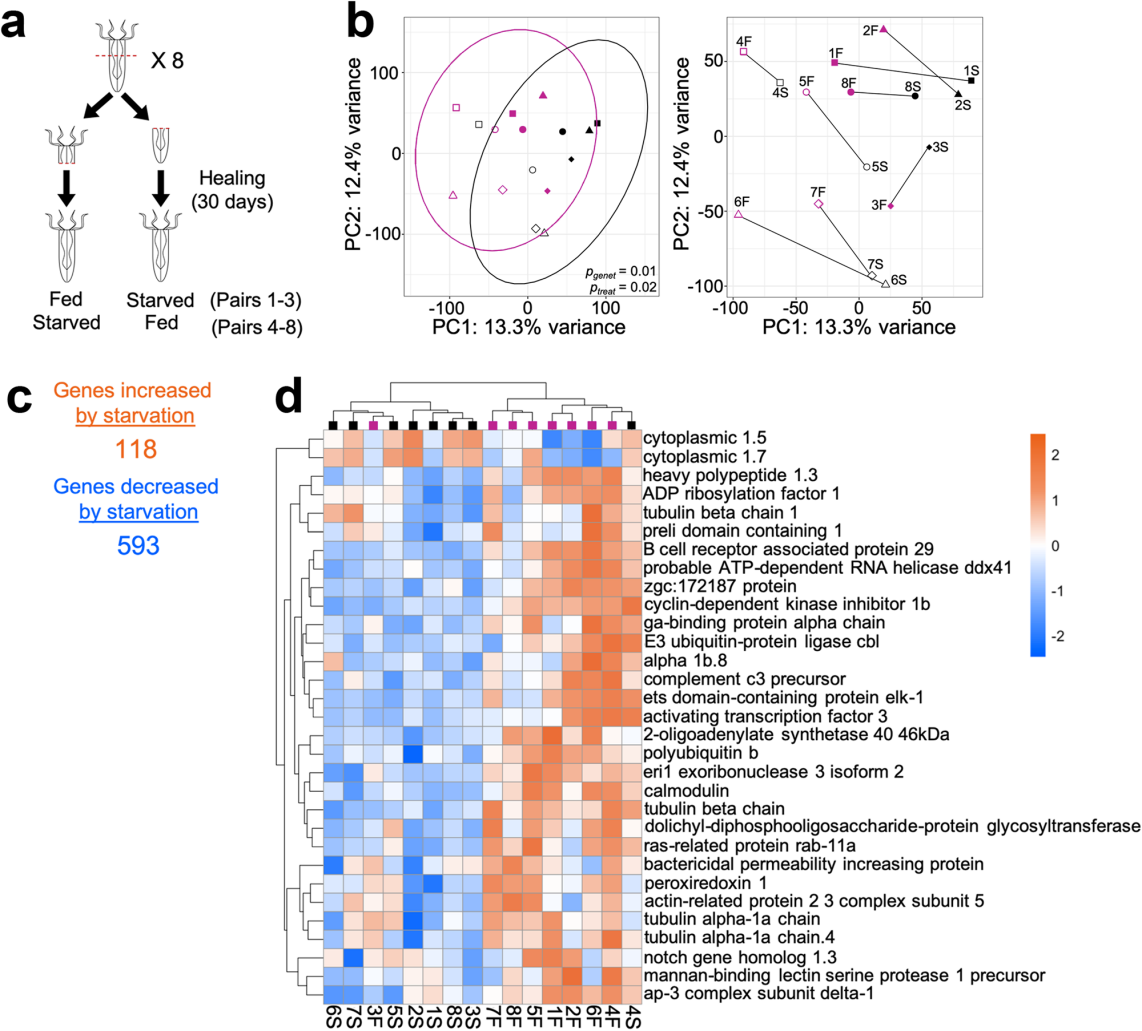
Changes in mRNA expression induced by starvation of adult *Nematostella*. **a** Schematic of experimental design. Eight adult anemones were bisected perpendicular to their body column and allowed to heal for 30 days before subjecting them to starvation. After 30 days of feeding or starvation, RNA was isolated and subjected to TagSeq analysis. **b** Principal component analysis (PCA) of *rlog* normalized gene expression profiles in *Nematostella*. Color denotes feeding status, i.e., either fed (pink) or starved for 30 days (black). Samples with the same symbol are clonal pairs. PERMANOVA results are shown for genetic background (*p*_*genet*_) and feeding treatment (*p*_*treat*_). PCA plot with ellipses (left) denoting multivariate t-distribution for both feeding status groups. The right shows the same plot with lines connecting samples that are clonal pairs. **c** Number of significantly (*FDR adjusted p* value < 0.1) increased and decreased DEGs after 30 days of starvation. **d** Hierarchical clustered heatmap of DEGs annotated with the “Immune Response” (*GO:0006955*) GO term and *FDR-adjusted p*-value < 0.1. Colors indicate the direction and the magnitude of the response based on the difference in expression relative to mean expression across all samples (blue, decreased expression; orange, increased expression). Colored squares at the top indicate feeding regime (pink, fed; black starved).

To assess the impact of reduced nutrition on *N. vectensis* (*Nv*), we performed genome-wide gene expression profiling using TagSeq on eight clonal adult anemone pairs wherein single animals from clonal pairs were either fed routinely or starved for 30 days prior to RNA isolation (Fig. 1a). Clonal pairs were used because preliminary experiments showed heterogeneity in protein expression when comparing anemones of differing genetic backgrounds. To avoid positional effects after regeneration, we included animals regenerated from aboral and oral ends in both fed and starved groups (Supplementary Table 1). Raw sequencing reads from the 16 anemones ranged from 5.2 to 9.2 million, and alignment values for all samples ranged from 74.2 to 77.4%.

To assess overall transcriptional differences between fed and starved anemones, *rlog-*normalized gene expression data were used for principal component analysis (PCA), which showed that nutritional status had a significant influence on gene expression (Fig. 1b, left; *Adonis* PERMANOVA *p*_*treat*_ = 0.02). The effect of genetic background (genet) also had a significant impact on gene expression (Fig. 1b, *Adonis* PERMANOVA *p*_*genet*_ = 0.01). These differences in gene expression between fed and starved animals were also seen when comparing individual clonal pairs; however, we note that each starved anemone showed a similar rightward shift along PC1 compared to its fed counterpart (Fig. 1b, right).

*DESeq2* identified 711 significant differentially expressed genes (DEGs) between starved and fed anemones while controlling for genetic background (FDR-adjusted *p*-value < 0.1): 118 genes were upregulated and 593 genes were downregulated in starved relative to fed anemones (Fig. 1c). A full list of the differentially expressed genes is presented in Supplementary Data 1.

### GO-based pathway responses to starvation

To investigate the biological response of adult *Nv* to starvation, a Gene Ontology (GO) enrichment analysis of “Biological Process” terms associated with significant DEGs (Supplementary Fig. 1) was performed using ranked *p* values. Among genes showing positive log-fold changes following starvation, three prominent Biological Process trends emerged: RNA processing (e.g., *RNA processing GO:0006396*, *RNA splicing GO:0008380*, *mRNA metabolic process GO:0016071*), DNA processing (e.g., *DNA metabolic process GO:0006259*, *DNA replication GO:0006260*, *DNA repair GO:0006281*), and chromosome organization (e.g., *chromatin remodeling GO:0006338*, *chromatin organization GO:0006325*, *covalent chromatin modification GO:0016569*).

Consistent with results from the DEG analysis (Fig. 1c) where nearly 85% of DEGs were downregulated following starvation, more GO terms were enriched among downregulated genes than among upregulated genes in starved anemones. Many GO terms enriched among downregulated genes reflected the nutrient-deprived status of the animals, with terms related to nutrient metabolism (e.g., *response to nutrient GO:0007584*, *lipid metabolic process GO:0006629, carbohydrate metabolic process GO:0005975*, *cellular amino acid metabolic process GO:0006520*) and cellular respiration (e.g., *electron transport chain GO:0022900*, *proton transmembrane transport GO:1902600*) (Supplementary Fig. 1). Thus, the GO terms enriched in downregulated genes indicate that the starvation regime that we used resulted in a transcriptional response consistent with metabolic suppression.

Of note, several immune-related terms were enriched among the downregulated genes following starvation (e.g., *immune response GO:0006955*, *defense response GO:0006952*, *antigen processing and presentation GO:0019882*), as well as some oxidative stress-related terms (e.g., *reactive oxygen species metabolic process GO:0072593*, *hydrogen peroxide metabolic process GO:0042743*) (Supplementary Fig. 1). Figure 1d presents expression data for the 31 DEGs in the *immune response* GO term that were both significantly downregulated (*FDR-adjusted p*-value < 0.1) and annotated. These 31 genes include *Nv* homologs of Notch and Elk1, and members of the complement system. Expression patterns of these genes clustered according to feeding regime, with the exception of the fed clone 3 and starved clone 4 (anemones 3F and 4S). Given that some individual anemone gene expression datasets did not cluster with the feeding regime (Fig. 1d) and to confirm that there were no labeling or processing mistakes, we analyzed single nucleotide polymorphisms (SNPs) in our TagSeq sequencing files and used them to assess the clonality of the analyzed anemone pairs. A total of 21,572 SNPs were found. Based on the analysis of SNPs, all individuals clustered with their assigned clonal pair (Supplementary Fig. 2). Thus, we believe that the variance seen with some anemones in our gene expression experiments (e.g., anemones 3F and 4S) reflects real diversity among individual anemones.

### Starvation increases the susceptibility of *N. vectensis* polyps to bacterial pathogenicity

**Fig. 2:**
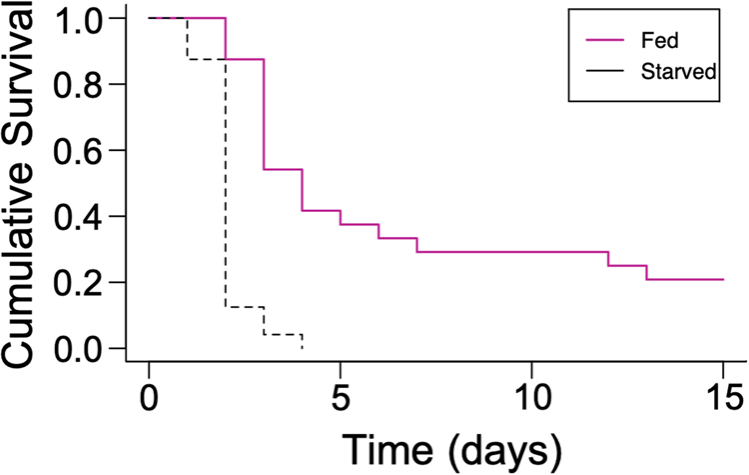
Starved anemones have increased susceptibility to *Pseudomonas aeruginosa* infection-induced death. 10-day-old anemones were either fed on a regular schedule (pink) for 30 days or starved (black), and were then infected with 6.8 × 10^8^ CFU/ml of *P. aeruginosa* at 28 ˚C. Survival was monitored daily for 15 days and recorded. *N* = 24 for both feeding regimes. Significance was determined using Kaplan-Meier statistics. These data are from experiment 2 in Supplementary Table 2.

Given that GO terms among downregulated DEGS in starved adult anemones were associated with immunity, we hypothesized that *Nv*’s ability to withstand bacterial challenge would be reduced in starved anemones. *Pseudomonas aeruginosa* is a Gram-negative bacterium that is pathogenic in a variety of hosts including some plants, invertebrates, and vertebrates^31,32^. Recently, *P. aeruginosa* strain PA14 was also shown to be pathogenic for juvenile *Nv*^28^. To determine whether fed and starved anemones have different susceptibilities to *P. aeruginosa*, we infected a total of 60 fed and 60 starved 40-day-old juvenile anemones over three separate experiments with an average of ~4.5 × 10^8^ CFU/ml of PA14. We then visually monitored disease progression daily based on tissue degradation and anemone death. We found that starved anemones died at a significantly faster rate than their fed counterparts (Kaplan-Meier *p* < 0.01, *N* = 24, Fig. 2; Supplementary Table 2), and that this pattern was reproducible across trials (Supplementary Fig. 3a, b and Supplementary Table 2 for two additional trials). Therefore, consistent with reduced expression of immunity-related genes in adults, starved juvenile *Nv* have increased susceptibility to the effects of a bacterial pathogen.

### NF-κB transcript and protein are reduced in starved anemones

Transcription factor NF-κB has a broad role in immunity across Metazoa^21,33,34^. We have previously shown that NF-κB protein is expressed in juvenile and adult *Nv*, with NF-κB protein expression being detectable as early as 30 h post-fertilization^26,35^. Given the upregulation of NF-κB following infection with *P. aeruginosa*^28^ and that several immunity-related genes were downregulated in our starved anemone gene expression dataset, we next sought to determine whether starvation had an effect on NF-κB. We first determined whether NF-κB transcripts were downregulated under starvation conditions in *Nv*. Gene expression comparisons between fed and starved adult *Nv* showed a trend towards reduced expression in starved anemones, based on two comparisons: 1) read-normalized NF-κB transcripts were lower in starved anemones in six of eight clonal pairs (average expression in starved anemones 0.65; Supplementary Table 3), and 2) a log_2_ Fold Change of −0.96 (log_10_, −0.51) by *DESeq2* analysis (Supplementary Data 1).

**Fig. 3:**
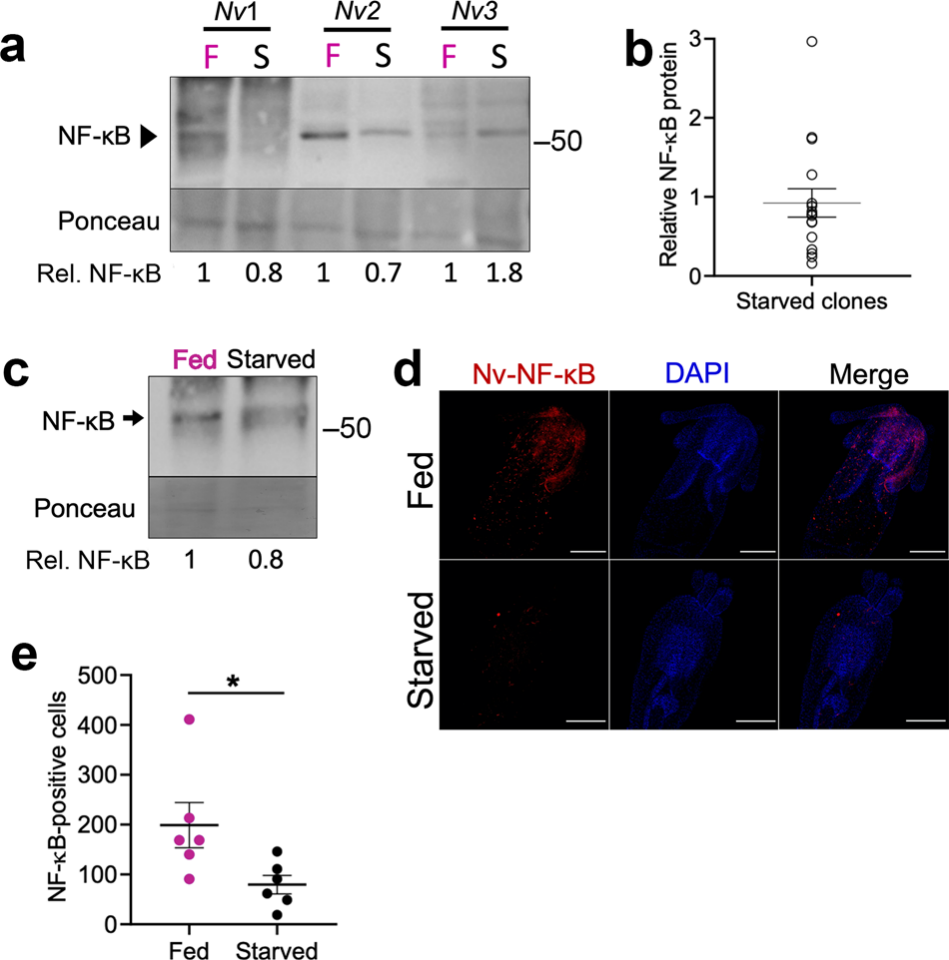
Starvation causes decreased levels of NF-κB. **a** 30-day fed or starved clonal *Nv* pairs were lysed and 100 µg of protein was electrophoresed on a 7.5% SDS-polyacrylamide gel and subjected to Western blotting with anti-Nv-NF-κB antiserum. NF-κB protein levels were normalized to Ponceau staining and quantified using ImageJ, and the relative (Rel.) NF-κB protein levels are indicated for each anemone pair. Molecular size markers in kDa are indicated to the right of the panel. Raw image is in Supplementary Fig. 8. **b** Scatter plot of relative levels of Nv-NF-κB protein after 30 days of starvation. 16 clonal pairs were fed or starved for 30 days and used for Western blotting and quantified as in (**a**). Each circle represents relative NF-κB for the starved anemone in one clonal pair. Horizontal line designates the mean of data. Error bars denote standard error. Raw images are in Supplementary Fig. 8. **c** Western blotting of juvenile anemones. Forty-day old *Nv* that had been either fed ground *Artemia* or never fed, were pooled together (100 individuals for each feeding regimen), lysed directly in SDS buffer, and lysates were subjected to Western blotting. Protein levels were normalized to Ponceau staining and quantified using ImageJ, and NF-κB protein level is relative (Rel.) to that seen in fed animals. Both naturally occurring alleles of Nv-NF-κB were accounted for during quantification. Molecular size markers in kDa are indicated to the right of the panel. Raw image is in Supplementary Fig. 8. **d, e** Whole-mount immunofluorescence was performed on 40-day old polyps that were either fed for 30 days or never fed. **d** Nv-NF-κB was detected with a custom antibody and visualized with Texas Red-labeled secondary antibody (red, left panels) and nuclei with DAPI (blue, middle panels). Scale bars, 100 μm. Images were taken on a Nikon C2 Si. Images are representatives of both feeding conditions. **e** Number of NF-κB-positive cells per feeding treatment (as denoted by circles). *N* = 6 for both treatments. Horizontal bars denote average for each group, and error bars denote standard error. Statistical significance (*) of difference of NF-κB-positive cells between feeding conditions was determined with an unpaired *t* test (*p* = 0.04). All raw microscopy images are in Supplementary Fig. 9, cell counts are in Supplementary Table 6.

We next compared NF-κB protein levels and DNA-binding activities in starved *vs* fed adult anemones. To do this, we again generated clonal pairs of anemones (by bisection and regeneration), which were then fed or starved for 14, 30, or 60 days. To compare NF-κB protein levels, Western blotting was performed on whole animal extracts from fed and starved clonal pairs. The 30-day starved adult anemones had less NF-κB protein than their fed counterparts in 12 of 16 anemones analyzed (Fig. 3a, b). Reduced NF-κB protein and DNA-binding activity were also seen in anemones that had been starved for 14 and 60 days (Supplementary Fig. 4). However, we wish to note that not all anemones showed reduced NF-κB protein levels at 14 or 30 days, a result that is consistent with the variability seen with the DEG and NF-κB transcript results (see Fig. 1d, above).

Because of the increased susceptibility of starved juvenile anemones to bacterial infection (Fig. 2), we were interested in determining whether the overall decrease in NF-κB protein during starvation was also observed in juvenile *Nv*. To do this, we performed anti-Nv-NF-κB Western blotting and immunohistochemistry on 40-day old *Nv* that had been fed regularly or never fed. To analyze NF-κB protein levels in juvenile *Nv*, we made separate pools of 100 fed and 100 starved 40-day-old anemones, lysed each pool by boiling in SDS sample buffer, and analyzed those lysates by Western blotting. Results showed that starved juvenile anemones had ~20% less NF-κB protein than fed anemones (Fig. 3c). In addition, starved juveniles had ~60% fewer detectable NF-κB-positive cells by immunohistochemistry (Unpaired *t-*test *p* < 0.05) (Fig. 3d, e). Taken together, these results suggest that starvation causes an overall decrease of NF-κB transcript and protein in adult anemones, which results in decreased DNA-binding activity, and that decreased average NF-κB protein levels are also seen in juvenile anemones, which have increased susceptibility to bacterial infection.

### Gene co-expression network analysis reveals possible cnidarian immune gene network

**Fig. 4:**
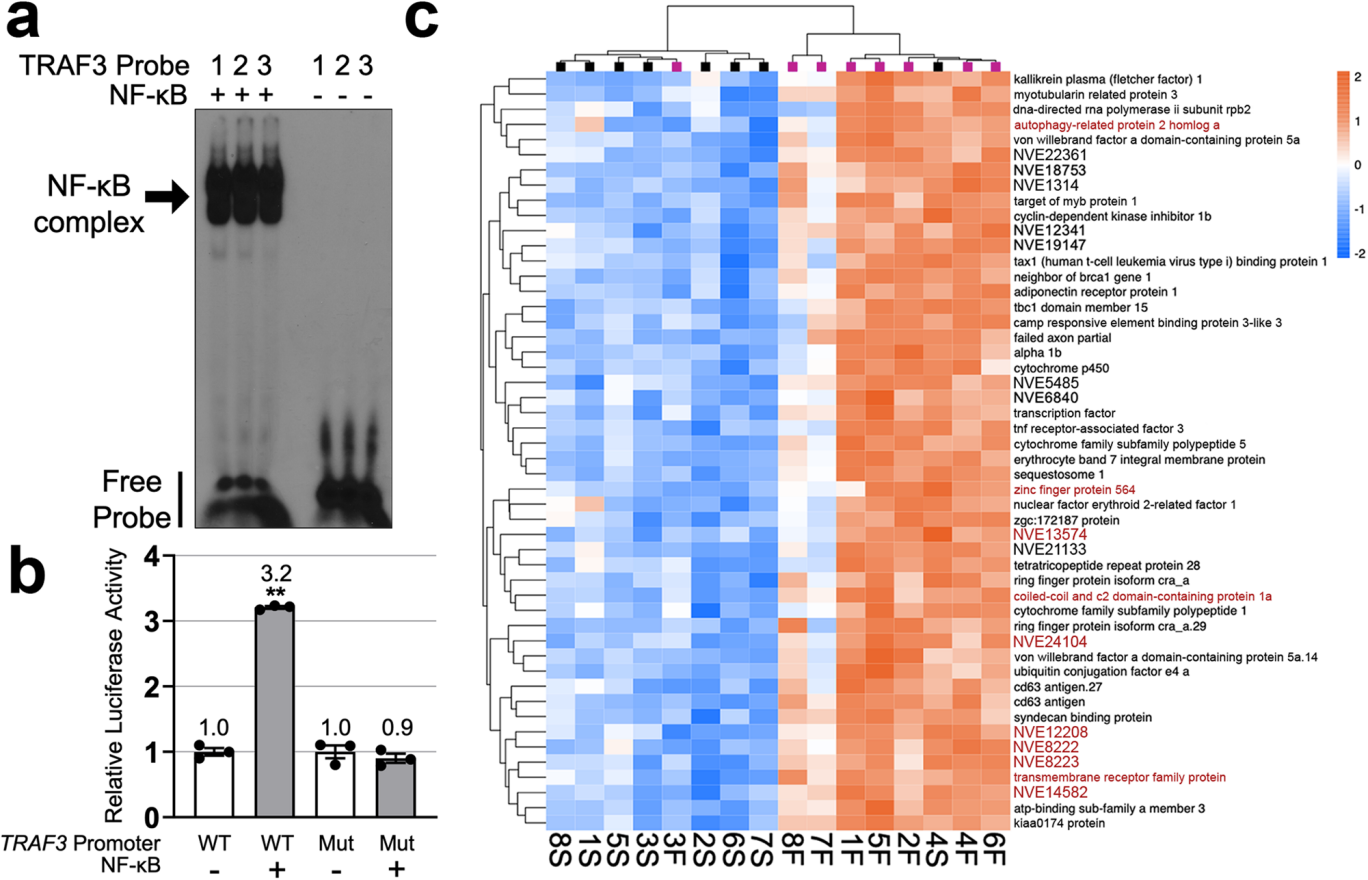
Nv-NF-κB gene expression network analysis. **a** Nv-NF-κB is able to bind three predicted κB-binding sites (1, 2, 3; see Supplementary Fig. 5) upstream of the *Nv* *TRAF3* TSS. Bacterially expressed GST-Nv-NF-κB (+) was incubated with ^32^P-labeled probes containing each predicted NF-κB-binding site from the *Nv* *TRAF3* promoter and analyzed by EMSA. Labeled probes alone (-) were used as negative controls. Raw image of the gel is in Supplementary Fig. 10. **b** Nv-NF-κB can activate an *Nv TRAF3* promoter luciferase reporter. HEK 293 cells were co-transfected with either an expression plasmid for Nv-NF-κB or the empty vector and a luciferase reporter containing either the wild-type *TRAF3* promoter (*TRAF3*-WT) or a mutant *TRAF3* promoter (*TRAF3*-Mut) in which all three NF-κB sites were mutated. Values are averages of triplicate individual samples (shown by circles) and are presented as values relative to the respective empty vector control. Error bars indicate standard error. (**) indicates statistical significance (*p* > 0.05) as determined using the Student’s *t test*. Raw luciferase values are in Supplementary Table 7. **c** Hierarchical clustered heatmap of “Green” module’s top 50 genes ranked by membership score (kME). Colors indicate direction and magnitude of expression compared to the mean expression across all samples (blue, decreased expression; orange, increased expression). Colored squares at the top indicate feeding regime (pink, fed; black starved). Genes highlighted in red have one predicted Nv-NF-κB-binding site within 500 bp upstream of their TSS (see Supplementary Table 4).

NF-κB is well-known for its role as master regulator of immunity^33,34^, and this role also includes the regulation of genes encoding other members of the NF-κB signaling pathway. One such member, TNF receptor-associated factor 3 (*TRAF3*), has been previously shown to have mRNA expression levels that are positively correlated with NF-κB expression in *Nv*^28^. In addition, we found that the *Nv TRAF3* gene has three predicted strong NF-κB binding sites within 1000 bp of its transcription start site (TSS) (Supplementary Fig. 5). All three of these predicted NF-κB sites can be bound by bacterially expressed Nv-NF-κB in an EMSA (Fig. 4a). Furthermore, a luciferase reporter plasmid containing the upstream promoter region of *Nv*-*TRAF3* can be activated by Nv-NF-κB when co-transfected in HEK 293 cells (Fig. 4b), and mutation of the three NF-κB binding sites abolished the ability of the reporter plasmid to be activated by Nv-NF-κB (Fig. 4b).

To gain a broader overview of a possible Nv-NF-κB gene expression network that is relevant to the starvation response, we used a systems biology-based approach wherein we identified modules of genes whose expression profiles were correlated with Nv-NF-κB mRNA expression. For this analysis, we used the R package *WGCNA* (Weighted Gene Correlation Network Analysis). This approach generated module eigengenes, or representative gene expression profiles for each module, and we identified the module containing NF-κB (“Green” module). This “Green” module, which contained 1317 genes, had a correlation coefficient of −0.66 with starvation (Supplementary Fig. 6), indicating that expression of genes in this module tended to be downregulated by starvation. We also found that 195 genes (15%) belonging to the “Green” module were significant DEGs as determined by our *DESeq2* analysis. We next ranked these transcripts by “membership score” (kME), which is a measure of how strongly each gene corelates with the module’s eigengene. Figure 4c shows a heatmap of the 50 genes in the “Green” module with the highest membership scores. These 50 genes (36/50 annotated) included ones encoding a *TRAF3* homolog and autophagy-related protein 2 homolog A, among others.

To further characterize the genes in the “Green” module, we performed a binary analysis of GO enrichment and found enrichment for terms related to Biological Processes including immunity (e.g., *immune response, regulation of immune response GO:0050776, antigen processing and presentation*), cell signaling (e.g., *regulation of IKK/NF-κB signaling GO:0043122, JNK cascade GO:0007254, MAPK cascade GO:0000165*), and cell death (e.g., *cell death GO:0008219, negative regulation of cell death GO:0060548, regulation of necrotic cell death GO:0010939*) (Supplementary Fig. 7). Additionally, we found that 10 of these top 50 genes (20%) had predicted Nv-NF-κB binding sites within 500 bp of their TSS (Fig. 4c, Supplementary Table 4). Of note, these predicted Nv-NF-κB binding sites in the upstream regions of these ten genes are high affinity sites, based on their high z-scores (Supplementary Table 4) from our previous analysis of DNA binding site preference for Nv-NF-κB using protein-binding microarrays^30^. As a control, we randomly chose 200 genes and searched the 500 bp upstream of their TSS for NF-κB binding sites. Sixteen genes (8%) in this random group had putative NF-κB binding sites (Supplementary Table 5). Thus, the top 50 “Green” genes were 2.5-fold more likely to have predicted NF-κB binding sites in their proximal upstream regions as compared to a random gene set (two sample Z test *p* = 0.01).

## Discussion

Here, we demonstrate a correlation between decreased nutritional status and decreased immunity in the sea anemone *Nematostella vectensis* (*Nv*). We have found that feeding status has a significant impact on gene expression, in addition to the effect of genetic background, consistent with what is seen in other cnidarian gene expression studies involving clonal populations^36,37^. Furthermore, we show that the level and activity of immunity-related transcription factor NF-κB are also generally reduced under starvation conditions. Thus, we demonstrate a link between nutritional status and immunity in a cnidarian, suggesting that a nutrition-immunity axis has broad evolutionary relevance. These results also have implications for other cnidarians, e.g., corals, which are endangered by rapidly changing environmental conditions.

Gene expression data and GO enrichment analysis provide insight into the transcriptional effects of starvation in our cnidarian model. Terms such as *response to nutrient*, *lipid metabolic process*, *carbohydrate metabolic process*, *glycosylation GO:0070085*, and *proteolysis GO:0006508* were enriched among downregulated genes under starvation conditions, which suggests an exhaustion of energetic sources. Downregulation of genes associated with metabolism of different energy sources (carbohydrates, lipids, proteins) during periods of starvation has been previously observed in other species^4,38–42^. Enrichment of these GO terms among downregulated genes in the starved anemones indicates that the anemones in our experiments were indeed responding in a manner consistent with nutritional deprivation. Moreover, GO terms among the downregulated genes including *ATP biosynthetic process GO:0006754*, *proton transmembrane transport*, and *electron transport chain GO:0022900* also support the energetic shortage experienced by *Nv* under starvation. Generally, animals that encounter prolonged periods of food deprivation exhibit low metabolic rates^1^, and so it is perhaps not surprising that our data showed downregulation of metabolic processes under food limitation.

In starved adult anemones, we also found a significant enrichment of downregulated genes and GO terms that are associated with immunity (e.g., GO terms *immune response*, *defense response*, and *antigen processing and presentation*). Additionally, we found that downregulated genes were enriched for GO terms associated with response to oxidative stress (e.g., *reactive oxygen species metabolic process*, *hydrogen peroxide metabolic process*), suggesting a vulnerability to oxidative stress. Oxidative stress has been shown to occur in vertebrates in response to prolonged starvation^43–45^, which further highlights the similarities of the response to starvation across metazoans.

Consistent with the DEG and GO term analyses, starved juvenile *Nv* had a statistically reduced ability to withstand infection by *P. aeruginosa*, which was correlated with an overall decrease in NF-κB protein levels, as judged by both Western blotting and immunostaining. This relationship between starvation and susceptibility to pathogen infection has been observed in invertebrates^3–5,11^, mice^7,9^, and humans^8,10^. For example, food limitation has been shown to reduce immunity against bacteria in the caterpillar *Manduca sexta*^3^. A similar relationship has also been found in *Apis mellifera ligustica* (Italian honeybee), wherein dietary supplementation with an essential fatty acid improved their ability to withstand bacterial infection and resulted in transcriptional upregulation of the NF-κB pathway genes *Toll*, *Myd88*, and *Dorsal* (NF-κB homolog)^11^. We also observed an average reduction in Nv-NF-κB protein and DNA-binding activity due to starvation, as well as a downregulation of NF-κB transcripts. The underlying concept shared by all of the examples noted above, as well as our results with *Nv* herein, is that the immune response is an energetically demanding process, which has led to the evolution of proper resource allocation under different nutritional states. For example, *Drosophila* diverts energy from growth and nutrient storage when *Toll* signaling is activated^46^, and parasitic infection in *Bombus terrestris* (Bumblebee) becomes more virulent under low-nutrient conditions^47^. That being said, we found heterogeneity in the overall trends for starvation-induced effects on gene expression, NF-κB protein and mRNA expression, and response to bacterial infection, all of which suggest that there is variability among individuals within a given population of *Nematostella*. This innate diversity in gene expression likely helps protect populations from sudden environmental changes and stressors similarly to how genetic diversity is beneficial to the long-term survival of a species when facing environmental stress^48^.

Efficient resource allocation is a fundamental part of how an organism interacts with its environment and how it responds to stress. Many tropical reef building corals derive the majority of their energy from intracellular symbiotic algae^49^; however, this symbiotic relationship can be lost under a variety of stressors in a process known as coral bleaching^50,51^. Evidence suggests that feeding via heterotrophy is important for corals to mitigate bleaching in the face of warming oceans^17,18^. For example, the branching coral *Montipora capitata* shows enhanced recovery from bleaching compared to other corals by increasing the amount of carbon it acquires through heterotrophy^19^. Similarly, the coral *Pocillopora meandrina* incorporates more heterotrophic carbon when there is more food available^52^. Combined with the data presented herein, cnidarians appear to have complex and dynamic ways to respond to stress in the midst of poor nutrient availability.

In addition to bleaching, coral infectious diseases appear to be increasing, which could be due to environmental effects on immunity. Over the last 50 years, ~40 different coral diseases have been described^53^, with one recent source of concern being Stony Coral Tissue Loss Disease that is affecting Caribbean corals^54,55^. Previous work by our group showed that symbiosis with Symbiodiniaceae algae in the anemone *Exaptasia diaphana* is negatively correlated with anemone NF-κB levels, suggesting that the symbiotic state decreases its NF-κB-dependent immunity^30^. Thus, the survival of some cnidarians under certain environmental stressors appears to be linked to nutrition-based effects on immunity.

The phylum Cnidaria emerged ~700 million years ago^56^, and individual cnidarians have likely evolved unique genes as part of their immune systems^25,57^. By taking a systems biology approach, we were able to identify modules of genes that were highly correlated with starvation status in *Nv*. We also identified a module of genes to which NF-κB belongs that was composed of 1317 genes, of which ~30% are unannotated. Given that many genes in this module are associated with immunity, cell signaling, and cell death, it is likely that some unannotated genes within this same module play roles in immunity, such as being direct anti-microbial effectors. Moreover, we found that 11 of the top 50 genes in the same module as NF-κB had one or more predicted NF-κB-binding sites identified using a PBM-based Nv-NF-κB DNA-binding site motif^30^. These results provide avenues to explore novel basal immune gene interactions and are consistent with an evolutionarily conserved role of NF-κB in immunity-related gene regulation.

Previous work in *Nv* identified an unannotated anti-microbial gene that is upregulated in response to the immune stimulatory molecule 2′3′-cGAMP^28^, however, that anti-microbial gene was not downregulated by starvation in our experiments, suggesting that it is not a direct NF-κB target gene. In contrast, three lines of evidence suggest that *TRAF3* is a direct target of NF-κB in *Nematostella*: 1) there are three strong NF-κB binding sites located within 1000 bp of the *TRAF3* TSS (Supplementary Fig. 5; Fig. 4a), 2) Nv-NF-κB can activate a reporter locus containing the upstream promoter region of *TRAF3* and this activation requires the upstream NF-κB binding sites (Fig. 4b), and 3) *Nv* *TRAF3* is upregulated by activation of the c-GAS-STING pathway and increased NF-κB levels in *Nematostella*^28^. Of note, mammalian *TRAF3* is a regulator of NF-κB and has a broad role in B-cell immune activation and survival^58^. A correlation between NF-κB and TRAF3 expression has been reported in several other cnidarian studies. First, NF-κB and *TRAF3* are coordinately induced to high levels in the stony elkhorn coral *Acropora palmata* following acute heat stress^59^. Additionally, *TRAF3* has been suggested to play a role in coral heat stress response^60,61^, and has been proposed to be an NF-κB target gene in heat-stressed *E. diaphana*^62^. Therefore, our results provide a dataset to explore new gene network interactions, as well as leading to the identification of unannotated genes that are involved in the cnidarian immune system. These unannotated genes could be previously unknown immune effectors such as anti-microbial agents.

Overall, we show a link between nutrition and immunity in *Nematostella*, and suggest that transcription factor NF-κB plays a role in this relationship. Thus, these data provide a model for better understanding the interplay between nutrition and immunity in cnidarians on a genetic and protein level. The continued study of these important pathways in early diverging lineages of metazoans will further our understanding of where and how these pathways originated, as well as having implications for their physiological effects in critical marine organisms as we move into an era of changing climate. Although *Nv* is a long-standing model organism for Cnidaria, transcriptomic and molecular tools are expanding for other cnidarians and early diverging lineages of invertebrates. Similar studies in other cnidarians will likely show whether the response to starvation is conserved among cnidarians and other invertebrates. The pathways through which *Nv* detects starvation and how they communicate to result in diminished NF-κB levels may also inform us about the evolution of mammalian immune systems.

## Materials and methods

### Care, husbandry, and cloning of *Nematostella vectensis*

*N. vectensis* from a Maryland population were obtained from Mark Martindale and Matt Gibson. Spawnings were performed as previously described^12,26,63,64^. Briefly, spawning in males and females was induced by transferring anemones into a 25 °C incubator with bright fluorescent light and incubating the animals overnight. The next day, eggs and sperm were collected, and fertilization was carried out by mixing the gametes.

Adults, polyps, and larvae were maintained in 1/3 strength artificial seawater (1/3 ASW: ~12 parts per 1000) in a dark incubator at 19 °C. Adult anemones were fed freshly hatched brine shrimp (*Artemia;* brineshrimpdirect.com*)* and young polyps were fed ground *Artemia* in 1/3 ASW three times per week. Water changes were performed weekly for all anemones. To generate clonal pairs, adult animals were allowed to fully relax and were then bisected perpendicularly to the oral/aboral axis. Halves were placed into separate wells of a 24-well plate, and anemones were allowed to regenerate for 30 days. Feeding was paused for all animals during the regeneration period and resumed once tentacles formed from the aboral end. Thereafter, both members of the clonal pair were fed in equal amounts.

### RNA extraction and preparation for TagSeq on fed vs. starved anemones

Clonal pairs of adult anemones were generated by bisection, and then the halves were allowed to heal for 30 days as described above. Thirty days was chosen to allow injury and regeneration-related genes to return to basal levels, as demonstrated previously^13,14^. Clonal pairs were fed equal amounts of food on a regular schedule during healing once all previously aboral ends had developed tentacles. Clonal pairs were split to be fed or starved for 30 days before being flash-frozen on dry ice prior to RNA extraction. Total RNA was isolated from eight clonal pairs with RNAqueous^TM^ Total RNA Isolation Kit (Invitrogen) according to the manufacturer’s instructions, with additional grinding using a plastic pestle during tissue lysis. Next, DNA was eliminated using DNA-*free*^TM^ DNA Removal Kit (Invitrogen). RNA quality was assessed by agarose gel electrophoresis, checking for the presence of ribosomal RNA bands. RNA concentrations were quantified using a NanoDrop ND-1000 Spectrophotometer. Samples were then normalized to 728 ng of total RNA for submission to the University of Texas at Austin—GSAF’s TagSeq Service. Libraries were created by the GSAF and sequenced on a NovaSeq 6000 SR100.

### Transcriptome read mapping

Reads were processed following the TagSeq pipeline (https://github.com/z0on/tag-based_RNAseq). In brief, adapters and poly(A)^+^ tails were trimmed using *Fastx_toolkit* and sequences <20 bp with <90% of bases having quality cut-off scores >20 were trimmed. PCR duplicates sharing degenerate headers were also removed. Resulting quality-controlled reads were aligned to the *Nematostella* transcriptome^65^ using *Bowtie2.2.0*^66^.

### Differential gene expression and gene ontology analyses

Differential gene expression analysis was performed using DESeq2 v.1.30.1^67^ in R v.4.0.4^68^. The *arrayQualityMetrics*^69^ package tested for outliers, which were defined as any sample failing two or more outlier tests; no outliers were identified. Significant DEGs were identified as those with an FDR-*adjusted p* value < 0.1. Expression data were normalized using the *rlog* function within the package *vegan*^70^, and normalized data were then used for PCA to characterize differences in gene expression between starved and fed (control) groups. Significance was tested by PERMANOVA using the *adonis* function as part of the *vegan* package^70^.

GO enrichment analysis was performed using Mann-Whitney *U* tests based on ranked *p* values^71^. GO enrichment results based on the “biological process” overarching division were plotted as dendrograms with GO categories clustering based on shared genes. Fonts and colors were used to indicate significance and direction of change respectively. Red was used for GO terms enriched in upregulated genes and blue for GO terms enriched in downregulated genes. To generate a heatmap of “Immune Response”-annotated genes, we used the package *pheatmap*^72^ to showcase differences in expression relative to mean expression across samples.

### Bacterial challenge of *N. vectensis*

Ten-day-old polyps were placed into single wells of a 24-well tissue culture plate, and they were then either fed ground-up *Artemia* for 30 days or starved until infection was initiated. Infection was performed essentially as described previously^28^. That is, single colonies of *P. aeruginosa* strain PA14 were cultured overnight in Luria Broth, bacteria were centrifuged for 10 min at 1627 × *g*, rinsed once with 1/3 ASW, centrifuged again, combined, and resuspended to an OD_600_ of ~0.1 in 1/3 ASW. A small aliquot was taken for plating to calculate CFU/ml. Polyps were infected by placing them in the well of a 24-well plate containing PA14 (1 ml). Survival was monitored daily, and mortality was determined based on tissue degradation and the absence of response to light and touch cues^27^. Infection was performed three separate times: once with 12 anemones per feeding regime, and twice with 24 anemones per treatment regime.

### Tissue lysis of *N. vectensis*

Whole protein lysates from anemones were generated following a protocol described previously^73^. Briefly, adult anemones (about 2-cm long) were homogenized using a plastic pestle in 1.5-ml microcentrifuge tubes containing 150 μl of ice-cold AT Lysis buffer with proteinase inhibitors (HEPES [20 mM, pH 7.9], EDTA [1 mM], EGTA [1 mM], glycerol [20% w/v], Triton X-100 [1% w/v], NaF [20 mM], Na_4_P_2_O_7_·10H_2_O [1 mM], dithiothreitol [1 mM], phenylmethylsulfonyl fluoride [1 mM], leupeptin [1 μg/ml], pepstatin A [1 μg/ml], aprotinin [10 μg/ml]). Cell lysis was enhanced by sonicating five times for 10 s on setting 3 with 1 min on ice in between, samples were then passed five times through a 30-gauge needle. NaCl was added to a final concentration of 150 mM. Finally, samples were centrifuged at 13,000 rpm for 30 min at 4 °C, and the supernatant was stored at −80 °C until needed. For protein lysates from juvenile polyps, hundred 40-day old polyps were pooled into a centrifuge tube. Seawater was removed by aspiration and 50 μl 4 × SDS sample buffer (Tris-HCl [0.25 M, pH 6.8], SDS [2.3% w/v], glycerol [10% w/v], β-mercaptoethanol [5% v/v], bromophenol blue [0.1% w/v]) was added to the tube. Samples were heated at 95 °C for 10 min with vortexing halfway through and at the end. Finally, 50 μl distilled H_2_O was added to the samples.

### Western blotting and electrophoretic mobility shift assay (EMSA)

Western blotting for Nv-NF-κB was performed according to methods developed previously^29,30,35,73^. Briefly, proteins were separated on a 7.5% SDS-polyacrylamide gel. Proteins were then transferred at 4 °C to a nitrocellulose membrane at 250 mA for 4 h and then 170 mA overnight. Nitrocellulose membranes were incubated in blocking buffer TBST (Tris-HCl [10 mM, pH 7.4], NaCl [150 mM], Tween 20 [0.1% v/v]) with powdered milk (5% w/v) (Carnation) at room temperature for 1 h. Membranes were then incubated in anti-Nv-NF-κB antibody^35^ (diluted 1:10,000 in blocking buffer) overnight at 4 °C. Membranes were washed several times with TBST before incubating with a horseradish peroxidase-conjugated anti-rabbit secondary antiserum (1:4000, Cell Signaling, #7074) for 1 h at room temperature. Membranes were then treated with SuperSignal West Dura Extended Duration Substrate (Pierce), and blots were imaged on a Sapphire Biomolecular Imager. The same filters were also stained with Ponceau S stain to ensure approximately equal total protein loading. To quantify bands in the Western blots, images were opened in ImageJ. Next, Nv-NF-κB bands were scanned using the Analyze Gel function to generate a value, and a portion of the corresponding Ponceau stained filter was also scanned to generate a protein loading value. The NF-κB value for each sample was obtained by dividing the raw NF-κB value by the protein loading (Ponceau) value. All values were then normalized to the NF-κB value for the corresponding fed clone (called 1.0).

EMSAs were performed according to methods developed prevriously^29,30,35,73^ using a ^32^P-labeled κB-site DNA probe (GGGAATTCCC) and adult anemone tissue lysates (described above). That is, lysates and ~200,000 cpm of the κB-site probe were incubated in binding buffer (HEPES [10 mM, pH 7.8], KCl [50 mM], DTT [1 mM], EDTA [1 mM], glycerol [4% w/v]) with poly dI/dC (40 ng), and BSA (200 ng) at 30 °C for 30 min. Supershifts were performed by incubating samples with 2 μl of anti-Nv-NF-κB antiserum, after binding to the DNA probe, for 1 h on ice. Samples were electrophoresed on a 5% polyacrylamide gel. EMSA gels were then dried and imaged on a Sapphire Biomolecular Imager (see Supplementary Fig. 11).

The EMSA for *TRAF3* promoter region was performed as above, except using bacterially expressed GST-Nv-NF-κB, which was purified using glutathione beads from bacterial lysates^30^. Purified GST-Nv-NF-κB was then incubated in binding buffer as described above with each of the following ^32^P-labeled probes:

(1) 5′-TCGAGAGGTCGGGAAAGCCCCCCCCCG-3′

(2) 5′-TCGAGAGGTCGGGAAACCCCCCCCCCG-3′

(3) 5′-TCGAGAGGTCGGGGAACTCCCCCCCCG-3′

Underlined sequences are predicted NF-κB binding sites in the *Nv TRAF3* promoter (Supplementary Fig. 5). The dried EMSA gel was exposed overnight to X-ray film at −80 °C overnight. Film was developed using a standard X-ray film developer.

### Immunohistochemistry of *N. vectensis* polyps

Immunohistochemistry was performed according to methods that we developed previously^26,35,73^. Polyps were fixed in formaldehyde (4%) in 1/3 ASW overnight at 4 °C and washed three times with PTx (Triton X-100 [0.2% v/v] in PBS). Antigen retrieval was done by microwaving samples in warm urea (5% w/v) at the lowest setting for 5 min. Samples were cooled at room temperature for 20 min. Samples were then washed three times with PTx. Samples were moved to blocking buffer (PTx + normal goat serum [5% v/v] + BSA [1% w/v]) and allowed to permeabilize overnight at 4 °C on a nutator. Blocking buffer was replaced with anti-Nv-NF-κB primary antiserum diluted in blocking buffer (1:100) and incubated overnight at 4 °C. Samples were then washed four times with PTx and incubated in Texas-red-conjugated anti-rabbit secondary antiserum (1:160, Invitrogen, #T-2767). Polyps were then washed four times with PTx. Nuclei were stained by adding DAPI to a final concentration of 5 mg/ml. Samples were imaged on a Nikon C2+ Si confocal microscope. NF-κB-positive cells were counted using the *Cell Counter* plug-in in ImageJ.

### Luciferase reporter gene assays

Luciferase reporter gene assays were performed in HEK 293 cells^35^. Cells were plated in six-well 35-mm plates to 60% confluence and transfected with: 0.5 μg of (i) pGL3-*Nv*-*TRAF3*, which consists of pGL3 with 1220 bp of the promoter region of *Nv*-*TRAF3* cloned upstream of the luciferase gene; or (ii) pGL3-*Nv*-*TRAF3*-3X-mut, similar to (i) but with all three putative κB-binding sites mutated to 5′-“GGGGAAAGCTT”-3′, and 2 μg of (i) a Nv-NF-κB expression plasmid or (ii) a pcDNA empty vector. Every transfection was performed with 15 μg of polyethylenimine. Two days after the transfection, cells were lysed with Reporter Lysis Buffer (Promega) following manufacturer’s instructions. Luciferase activity was measured using the Luciferase Assay System (Promega, #E397A) on cell lysates that contained equal amounts of protein. Luciferase activities for each triplicate were averaged and were then normalized to the empty vector control (set as 1.0). Values are reported as the averages of the three values plus standard error (SE), determined as follows:

1 $${{{{{\rm{SE}}}}}}={{{{{\rm{\sigma }}}}}}/{{{{{\rm{\surd }}}}}}n$$

where *σ* is the sample standard deviation, and *n* is the number of independent experiments.

### Weighted correlation network analysis (WGCNA)

Weighted Gene Correlation Network Analysis was performed using *WGCNA*^74^ and genes with low basemean values (<3) were removed and all remaining data were *rlog-*normalized. Outlier samples were checked within the *WGCNA* package, and no outliers were detected. Unsigned connectivity between genes was determined and eigengene expression of these modules were correlated to feeding conditions. The “Green” module was chosen by manually searching modules for the NF-κB transcript. GO enrichment analysis was performed as described in the “Differential Gene Expression Analysis” text with the modification of using Fisher’s exact test instead of ranked *p* values. To generate module heatmap, genes with the highest module membership scores (kME values) within specific modules (e.g., “Green” module) were identified and relative expression was plotted using the package *pheatmap*^72^.

To identify genes with putative NF-κB binding sites, the transcripts of the top 50 “Green genes” (Fig. 4c) were aligned to the *N. vectensis* genome^75,76^ on Ensembl^77^ using BLAST to identify genomic location. We then extracted 500 bp upstream of the TSS of every matched gene. To identify Nv-NF-κB binding sites in these upstream regions, we used the program FIMO^78^ (with a *p*-value cutoff of 7E-05) and a DNA site motif based on Nv-NF-κB DNA binding from a Protein Binding Microarray (PBM)^30^.

### Confirmation of *N. vectensis* clones through SNP analysis

To identify and confirm the clonality of anemone pairs in our dataset, we called SNPs from TagSeq reads for individual fed and starved anemones. Briefly, raw sequencing reads from the 16 anemones ranged from 5.2 to 9.2 million. *Fastx_toolkit* and sequences <20 bp with <90% of bases having quality cut-off scores >20 were trimmed. PCR duplicates sharing degenerate headers were also removed. Resulting quality-controlled reads were aligned to the *Nematostella* transcriptome^65^ using *Bowtie2.2.0*^66^. Resulting SAM files were converted to BAM files using *samtools*^79^. *ANGSD*^80^ was used to calculate pairwise identity-by-state (IBS) matrices using the following filters: minMapQ of 20, minQ of 25, dosnpstat of 1, sb_pval of 1e-5, hetbias_pval of 1e-5, skipTriallelic of 1, mindInd of 13, snp_pval of 1e-5, and minMaf of 0.05. IBS matrices were used as input for a custom R script, which visualized relatedness of individuals and allowed for the identification of clonal pairs of anemones.

### Statistics and reproducibility

Reporter gene assays in tissue culture cells (Fig. 4b) were performed with multiple independent samples as described in Materials and Methods and the figure legend, and the values are reported with standard error (as determined using Eq. 1). The sample sizes were as follows: Fig. 4b, *n* = 3. For PBM analysis, the median fluorescence values for each unique DNA-binding sequence were determined over eight replicate probe measurements. Log median fluorescence values were transformed into a “z-score” as described previously^30^. Other experiments, e.g., EMSAs, Western blots, were performed multiple times and had similar results to those reported here. Moreover, such experiments have internal controls for molecular weight markers and loading accuracy. Statistical analyses of DEGs, gene upstream regions (for predicted NF-κB binding sites), immunofluorescent staining of Nv-NF-κB, and Kaplan-Meier survival plots were performed as described above in Materials and Methods. Statistical analysis of immunofluorescent staining of Nv-NF-κB was analyzed as described in the legend to Fig. 3.

### Reporting summary

Further information on research design is available in the Nature Portfolio Reporting Summary linked to this article.

## Supplementary information

Supplementary Information

Description of Additional Supplementary Files

Supplementary Data 1

Reporting Summary

## Data Availability

The *Nematostella vectensis* transcriptome can be found at https://figshare.com/articles/dataset/Nematostella_vectensis_transcriptome_and_gene_models_v2_0/807696. High-resolution images of Supplementary Figs. 1 and 7 are available at https://github.com/joshuaguirre29/Nematostella_nutrition_and_starvation. Raw reads have been submitted to SRA under Accession Number PRJNA837630. Additional data relating to the study are available from the corresponding author upon reasonable request.

## Code availability

Protocols and scripts for TagSeq library preparation and analysis are available at https://github.com/z0on/tag-based_RNAseq. Protocol for Gene Ontology analysis is available at https://github.com/z0on/GO_MWU. Scripts for all other analysis used in this publication are available at https://github.com/joshuaguirre29/Nematostella_nutrition_and_starvation.
