# Supplementary material for: Starvation decreases immunity and immune regulatory factor NF-κB in the starlet sea anemone Nematostella vectensis
Source: Commun Biol. 2023 Jul 7;6:698. doi: 10.1038/s42003-023-05084-7 (PMC10329013; doi:10.1038/s42003-023-05084-7)
Supplement: Supplementary file 1 — Supplementary Information [file 42003_2023_5084_MOESM1_ESM.pdf]

Supplementary Information for

Starvation decreases immunity and immune regulatory factor NF-κB in the starlet sea anemone *Nematostella vectensis*

Pablo J. Aguirre Carrión, Niharika Desai, Joseph J. Brennan, James E. Fifer, Trevor Siggers, Sarah W. Davies & Thomas D. Gilmore*

*Thomas D. Gilmore
Email: gilmore@bu.edu

This file includes:

Supplementary Figures 1 to 11
Supplementary Tables 1 to 7
High-resolution of GO-term dendrograms publicly available at:
https://github.com/joshuaguirre29/Nematostella_nutrition_and_starvation

Other materials for this manuscript include the following:

Supplementary Data 1

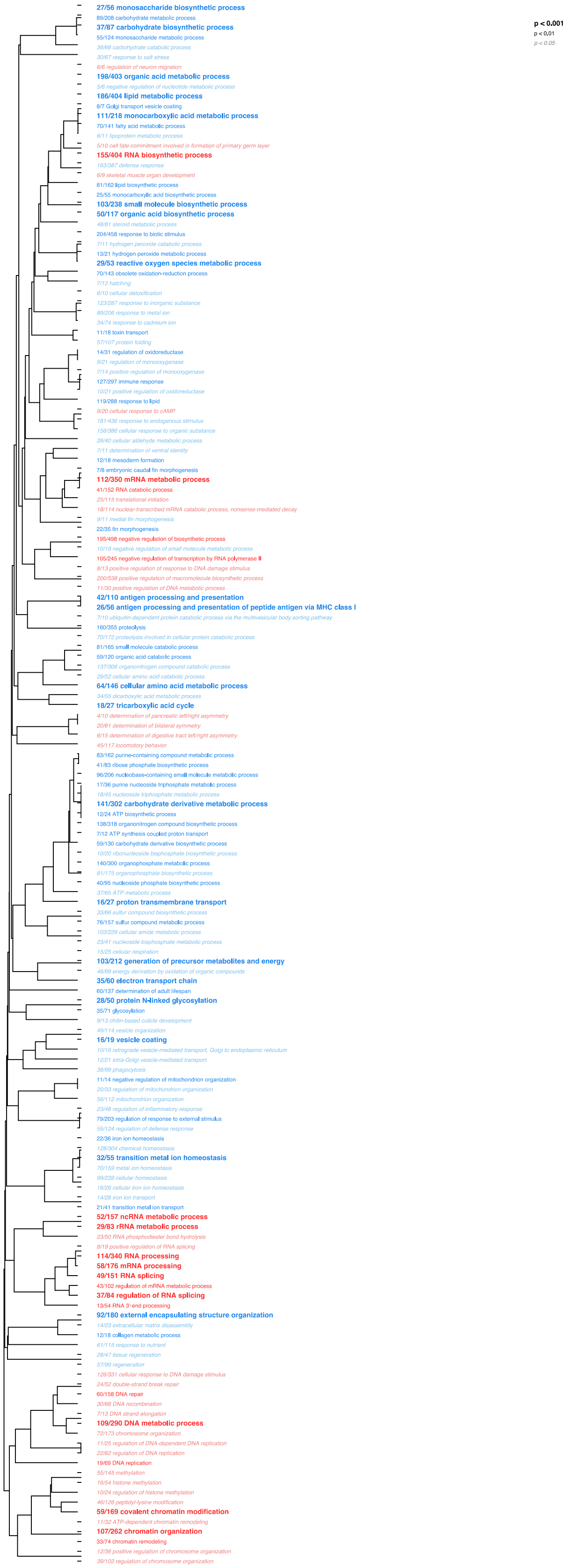
p < 0.001
p < 0.01
p < 0.05
27/56 monosaccharide biosynthetic process
89/208 carbohydrate metabolic process
37/87 carbohydrate biosynthetic process
55/124 monosaccharide metabolic process
36/68 carbohydrate catabolic process
30/67 response to salt stress
6/6 regulation of neuron migration
198/403 organic acid metabolic process
5/6 negative regulation of nucleotide metabolic process
186/404 lipid metabolic process
8/7 Golgi transport vesicle coating
111/218 monocarboxylic acid metabolic process
70/141 fatty acid metabolic process
6/11 lipoprotein metabolic process
5/10 cell fate commitment involved in formation of primary germ layer
155/404 RNA biosynthetic process
163/387 defense response
6/9 skeletal muscle organ development
81/162 lipid biosynthetic process
25/55 monocarboxylic acid biosynthetic process
103/238 small molecule biosynthetic process
50/117 organic acid biosynthetic process
48/81 steroid metabolic process
204/458 response to biotic stimulus
7/11 hydrogen peroxide catabolic process
13/21 hydrogen peroxide metabolic process
29/53 reactive oxygen species metabolic process
70/143 obsolete oxidation-reduction process
7/12 hatching
6/10 cellular detoxification
123/287 response to inorganic substance
89/206 response to metal ion
34/74 response to cadmium ion
11/18 toxin transport
57/107 protein folding
14/31 regulation of oxidoreductase
9/21 regulation of monooxygenase
7/14 positive regulation of monooxygenase
127/297 immune response
10/21 positive regulation of oxidoreductase
119/288 response to lipid
9/20 cellular response to cAMP
181/436 response to endogenous stimulus
156/386 cellular response to organic substance
26/40 cellular aldehyde metabolic process
7/11 determination of ventral identity
12/18 mesoderm formation
7/8 embryonic caudal fin morphogenesis
112/350 mRNA metabolic process
41/152 RNA catabolic process
25/115 translational initiation
18/114 nuclear-transcribed mRNA catabolic process, nonsense-mediated decay
9/11 medial fin morphogenesis
22/35 fin morphogenesis
195/498 negative regulation of biosynthetic process
10/19 negative regulation of small molecule metabolic process
105/245 negative regulation of transcription by RNA polymerase II
8/13 positive regulation of response to DNA damage stimulus
200/538 positive regulation of macromolecule biosynthetic process
11/30 positive regulation of DNA metabolic process
42/110 antigen processing and presentation
26/56 antigen processing and presentation of peptide antigen via MHC class I
7/10 ubiquitin-dependent protein catabolic process via the multivesicular body sorting pathway
160/355 proteolysis
70/172 proteolysis involved in cellular protein catabolic process
81/165 small molecule catabolic process
59/120 organic acid catabolic process
137/306 organonitrogen compound catabolic process
29/52 cellular amino acid catabolic process
64/146 cellular amino acid metabolic process
34/55 dicarboxylic acid metabolic process
18/27 tricarboxylic acid cycle
4/10 determination of pancreatic left/right asymmetry
20/61 determination of bilateral symmetry
6/15 determination of digestive tract left/right asymmetry
45/117 locomotory behavior
83/162 purine-containing compound metabolic process
41/83 ribose phosphate biosynthetic process
96/206 nucleobase-containing small molecule metabolic process
17/36 purine nucleoside triphosphate metabolic process
18/45 nucleoside triphosphate metabolic process
141/302 carbohydrate derivative metabolic process
12/24 ATP biosynthetic process
138/318 organonitrogen compound biosynthetic process
7/12 ATP synthesis coupled proton transport
59/130 carbohydrate derivative biosynthetic process
10/20 ribonucleoside bisphosphate biosynthetic process
140/300 organophosphate metabolic process
81/175 organophosphate biosynthetic process
40/95 nucleoside phosphate biosynthetic process
37/65 ATP metabolic process
16/27 proton transmembrane transport
33/66 sulfur compound biosynthetic process
76/157 sulfur compound metabolic process
103/229 cellular amide metabolic process
23/41 nucleoside bisphosphate metabolic process
15/25 cellular respiration
103/212 generation of precursor metabolites and energy
46/99 energy derivation by oxidation of organic compounds
35/60 electron transport chain
60/137 determination of adult lifespan
28/50 protein N-linked glycosylation
35/71 glycosylation
9/13 chitin-based cuticle development
49/114 vesicle organization
16/19 vesicle coating
10/16 retrograde vesicle-mediated transport, Golgi to endoplasmic reticulum
12/21 intra-Golgi vesicle-mediated transport
38/99 phagocytosis
11/14 negative regulation of mitochondrion organization
20/33 regulation of mitochondrion organization
56/112 mitochondrion organization
23/48 regulation of inflammatory response
79/203 regulation of response to external stimulus
55/124 regulation of defense response
22/36 iron ion homeostasis
128/304 chemical homeostasis
32/55 transition metal ion homeostasis
70/159 metal ion homeostasis
99/239 cellular homeostasis
16/26 cellular iron ion homeostasis
14/28 iron ion transport
21/41 transition metal ion transport
52/157 ncRNA metabolic process
29/83 rRNA metabolic process
23/50 RNA phosphodiester bond hydrolysis
8/19 positive regulation of RNA splicing
114/340 RNA processing
58/176 mRNA processing
49/151 RNA splicing
43/102 regulation of mRNA metabolic process
37/84 regulation of RNA splicing
13/54 RNA 3'-end processing
92/180 external encapsulating structure organization
14/23 extracellular matrix disassembly
12/18 collagen metabolic process
61/115 response to nutrient
28/47 tissue regeneration
57/99 regeneration
126/331 cellular response to DNA damage stimulus
24/52 double-strand break repair
60/158 DNA repair
30/68 DNA recombination
7/13 DNA strand elongation
109/290 DNA metabolic process
72/173 chromosome organization
11/25 regulation of DNA-dependent DNA replication
22/62 regulation of DNA replication
19/69 DNA replication
55/145 methylation
16/54 histone methylation
10/24 regulation of histone methylation
46/126 peptidyl-lysine modification
59/169 covalent chromatin modification
11/32 ATP-dependent chromatin remodeling
107/262 chromatin organization
33/74 chromatin remodeling
12/36 positive regulation of chromosome organization
39/102 regulation of chromosome organization

Supplementary Fig. 1 Gene Ontology (GO) analysis of significantly enriched ‘Biological Process’ terms in starved anemones. Mann-Whitney U tests (GO-MWU) was used based on ranked p-values. Dendrograms clusters terms based on genes shared between categories. Color indicates enrichment in starved anemones: blue indicates terms enriched in downregulated genes, and red indicates terms enriched in upregulated genes. The p-values are designed by font size as indicated at the top right.

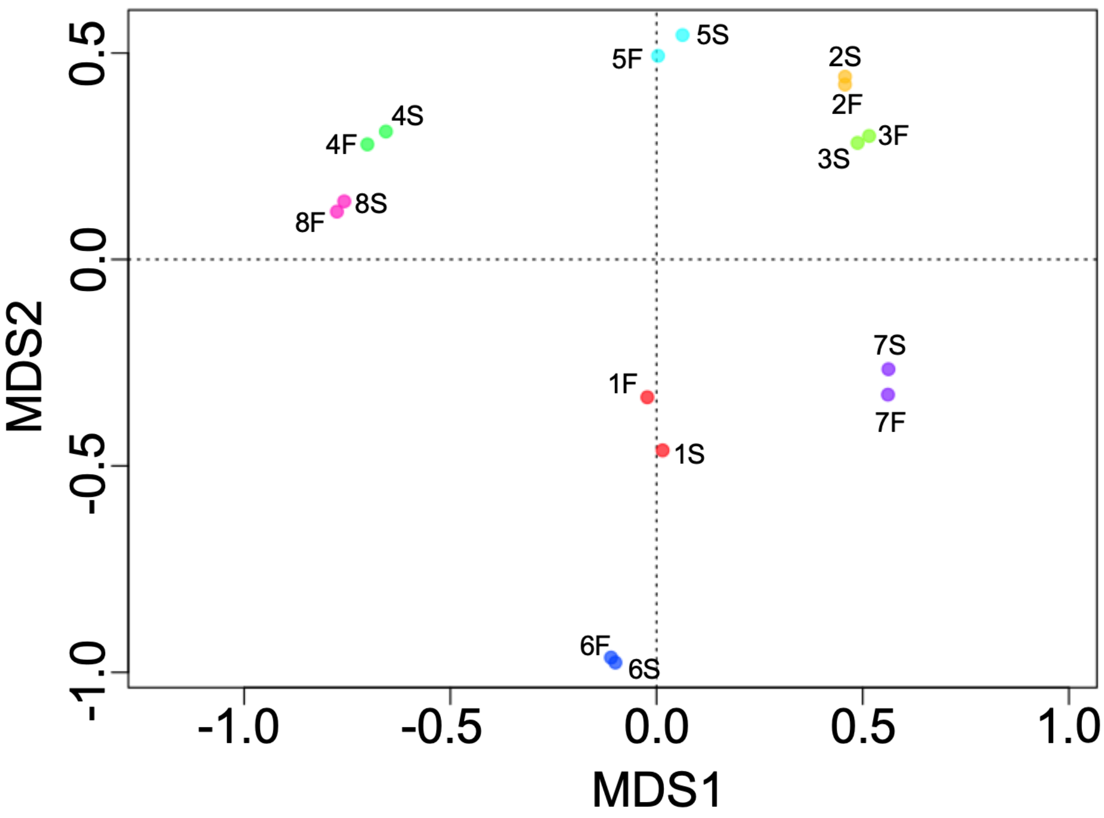

Supplementary Fig. 2 Single nucleotide polymorphism (SNP) analysis of starved and fed clonal pairs of anemones. Multidimensional scaling (MDS) plot based on *ANGSD*-calculated pairwise identity-by-state matrices of all samples. 21,572 SNPs were identified and used to determine clonality. Matching colors indicate clonal pairs.

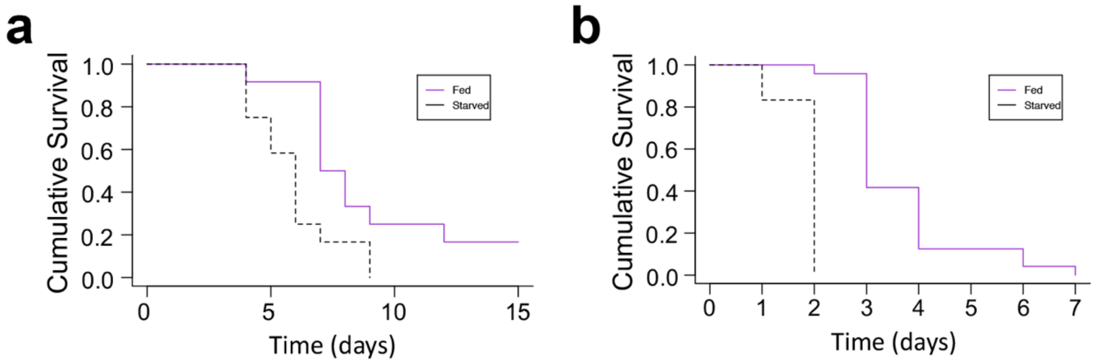

Supplementary Fig. 3 Starved anemones have increased susceptibility to *Pseudomonas aeruginosa* infection-induced death. Two-week old anemones were either fed on a regular schedule (purple) for 30 days or starved (black), and were then infected with **a,** 4.4 x 10^8 or **b,** 2.5 x 10^8 CFU/ml of *P. aeruginosa* at 28˚C. Survival was monitored daily for 15 days and recorded. **a,** N = 12 and **b,** N = 24 for each condition. Significance was determined using Kaplan-Meier statistics.

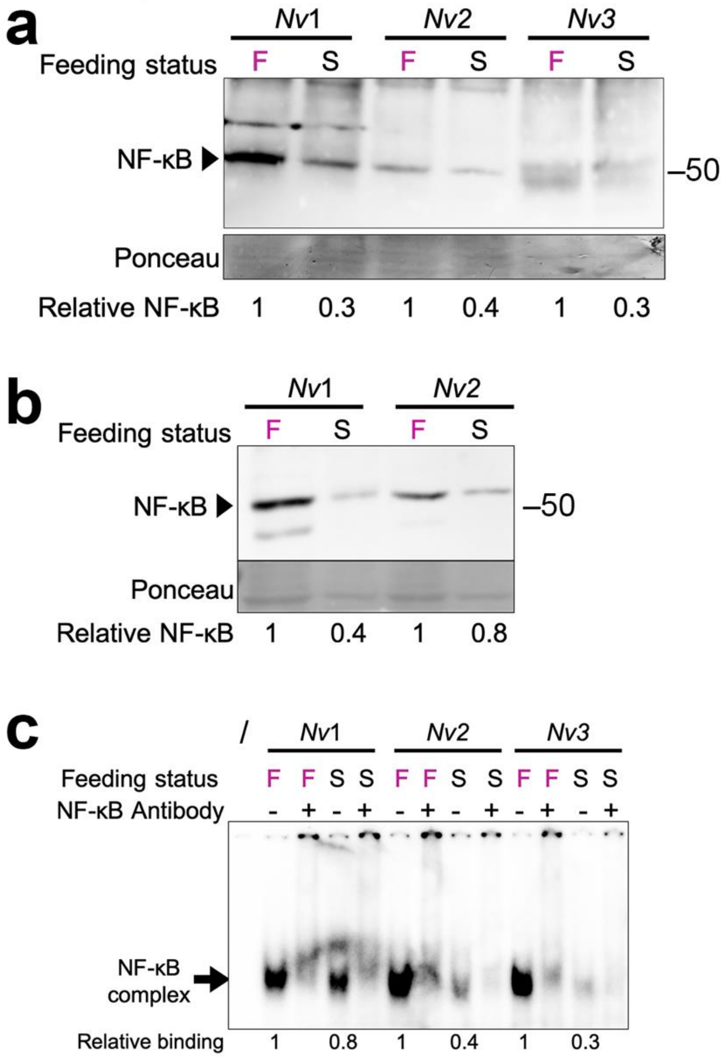

Supplementary Fig. 4 Starvation leads to decreased levels of NF-κB protein and DNA binding. a, 14-day and **b,** 60 day fed or starved clonal *Nv* pairs were lysed and 100 µg of protein was electrophoresed on a 7.5% SDS-polyacrylamide gel and subjected to Western blotting with anti-Nv-NF-κB antiserum. NF-κB protein levels were normalized to Ponceau staining and quantified using ImageJ, and the relative NF-κB protein levels are indicated for each anemone pair. Molecular size markers in kDa are indicated to the right of the panel. **c,** Starvation decreases Nv-NF-κB DNA-binding activity. 14-day fed or starved clonal *Nv* pairs were lysed and 100 µg of protein was incubated with a ^{32}P-labelled κB probe (GGGAATTCCC), and samples were then analyzed by EMSA. Feeding status is labeled as follows: F, fed and S, starved. Where indicated, samples were also incubated with anti-NF-κB antiserum for super shifting. First lane denoted by '/' is the probe with no protein extract. Raw images of the gels in all panels of this figure are in Supplementary Fig. 11.

```
>TRAF3 promoter
GTGTTTGGGAAAAGTGACAGCGACTTTAGTTTTCGCATGATGTACCGTGGCGGCGTGTGC
AACAGGGAAAGCCCCCAGATCCTTTATTGATGATCAAACTCACATGGGCAATAGGCGTGG
GTGGATACGGTGATTTTTTGCTGTGCCAAATAACTAGCAATTGTGTGTGAATATAATTTA
CAACTAGTCCCGTGTCGCAATTGAATACTATTTTGGTATTTTGACTCGCAGGATCTTCTG
GGTCAGTACGCAGCTCTTACAAGCTGCAATTTGATTGGGCAAATGAACAATATCCGCACC
TTGACACAAAGAACGAGAAGAATAGAGACAAAAGAACTCCTTTGTAGAACAAAGATAATA
GGAGACAAAGCAGCTGCGTGCTTACTCAGATCATTGCAAGATGTCACAACAATGAAAAAA
TCAACAGGTTGTGCATATTTCAGCATTACTTGAATGAAAAAATAATGAGAAAATGATGAA
AGCGCACCGAGGGGGCGATGAGTTTCATAAATACTACTTCTACTCTCTATTGTCAAAATC
TTATAATATTTTGAAGGAAACCGGTGCGTCAAAATCGTTTTTAAATAAAACAAATGGATT
AGACAACAATGGTACCCTCAAAGCCAGATAGGATATATCTAAAACTATATTTATTAGTGT
GTTTCCAAAACCAATTAAAAGATTCATGCATACATTGTAATAAACCGAGAAGACTACAAC
GATAATGCACTAATCGTCACATTACGCAAATGTGAAAGGAAAAACCGAGGAACGAACCCT
TTTCGCATTCCATTTTGATCTGTTACAAGAGCAGATAGTAAAACATTTGCCTGTTTCGAG
CTGCTTGGAACGATTGGCACAGGACGATTGATTAAGCAGCTTGCGTTACAAAGAGCACAG
AGCAGTTATTGTCAGGTACATGTAGCGATAGCAGGGAAACCCCCTCTTCACAGTCCTATA
CGATTGGTCGAGGTCATGATTGCGAACTATTCGGGGAACTCCCGTATGCTAAGTTCTTTC
ACTTTCGCTTTTAATCAGAGTTTTTGGCGCAAAGTTTCGGCAGTATGAGTCTCGATAGTA
CTAGGTTTGAATATATCGATGTTCTCGAGGATAGATACATCTGCAACGCATGTAGAATGC
CGCTGAACCATCCAATGCAGGCTCAGTGTGGACACAGGTTGGTACAAAACATTGAAAGGG
```

Supplementary Fig. 5 The *Nv TRAF3* proximal promoter region. Predicted NF-κB binding sites are highlighted in red. The three sites are numbered 1, 2, and 3 from top to bottom. These sites had PBM-based DNA-binding z-scores as follows: site 1, 6.1; site 2, 9.7; and site 3, 6.9 (see ref.[29] for details). The transcription start site is highlighted in green and underlined.

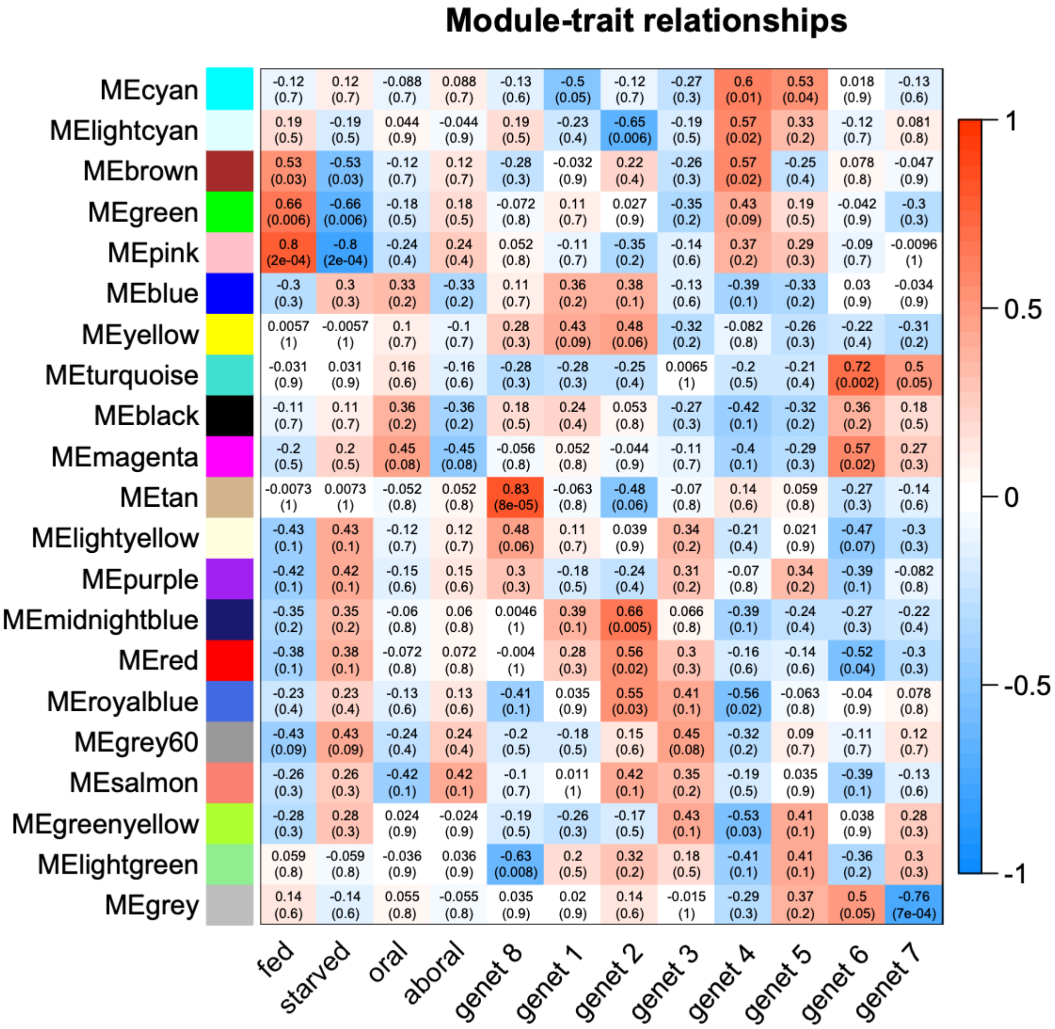

Supplementary Fig. 6 WGCNA-generated heatmap of traits and gene modules. WGCNA was performed using the package *WGCNA*. Color and shading correspond to correlation between trait (bottom) and gene module (left), positive numbers (red) indicate positive correlation, negative numbers (blue) indicate negative correlation between modules and traits. ‘fed’ and ‘starved’ indicate feeding status; ‘oral’ and ‘aboral’ indicate whether regenerated individuals originated from oral or aboral end; genets 1-8 indicate clonal genotype.

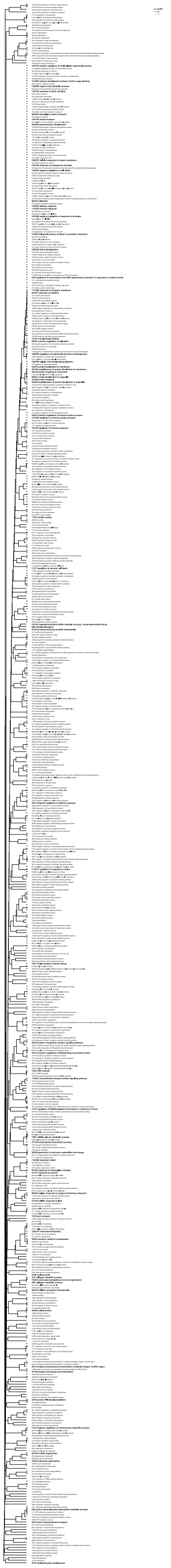

Supplementary Fig. 7 Gene Ontology (GO) of significantly enriched 'Biological Process' terms in WGCNA-generated gene module containing NF-κB using Mann-Whitney U tests (GO-MWU) based on continuous kME. Dendrogram clusters terms based on genes shared between categories. Shown are Biological Process terms that are enriched in the green module (containing NF-κB). The p-values are designated by the font as shown in the upper right.

Fig. 3a

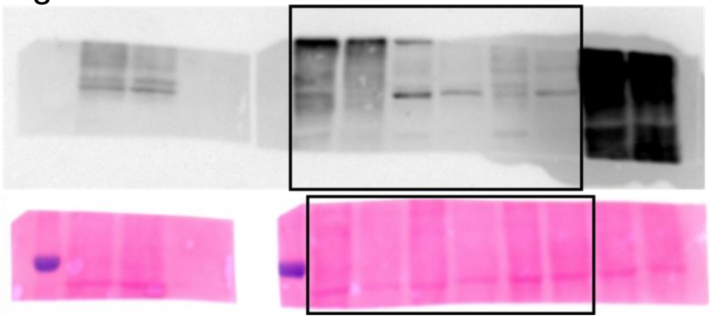

Fig. 3b

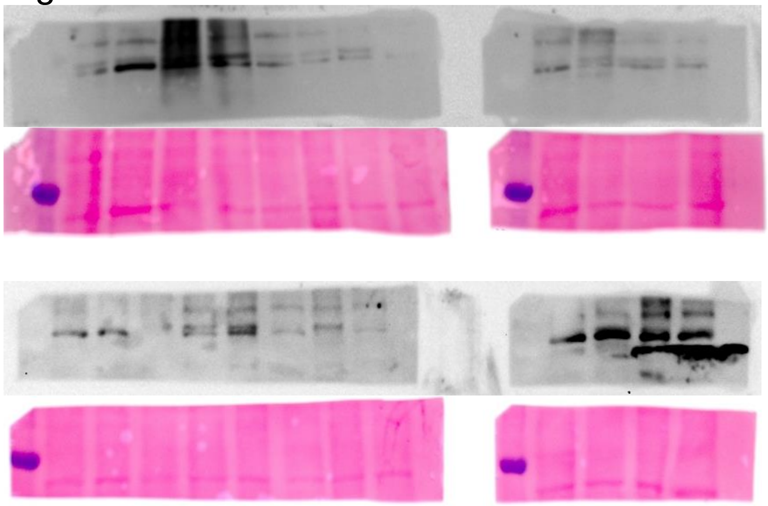

Fig. 3c

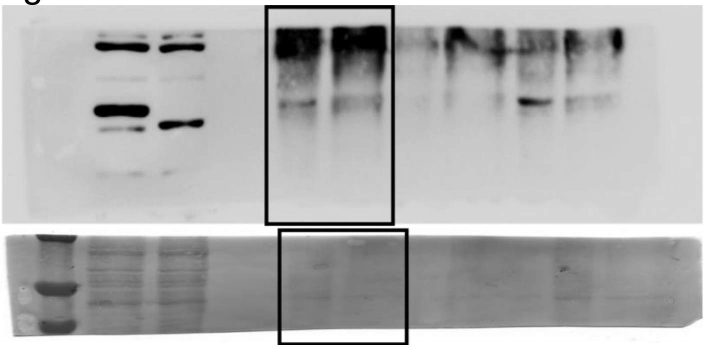

Supplementary Fig. 8 Uncropped versions of gels for Figs. 3a, 3b, and 3c. In Fig. 3a, the boxed areas were used to create Fig. 3a in the text. The lanes in Figs. 3a and 3b were used to obtain the data points for the graph in Fig. 3b. The boxed lanes in Fig. 3c were used to create Fig. 3c in the text.

Fed

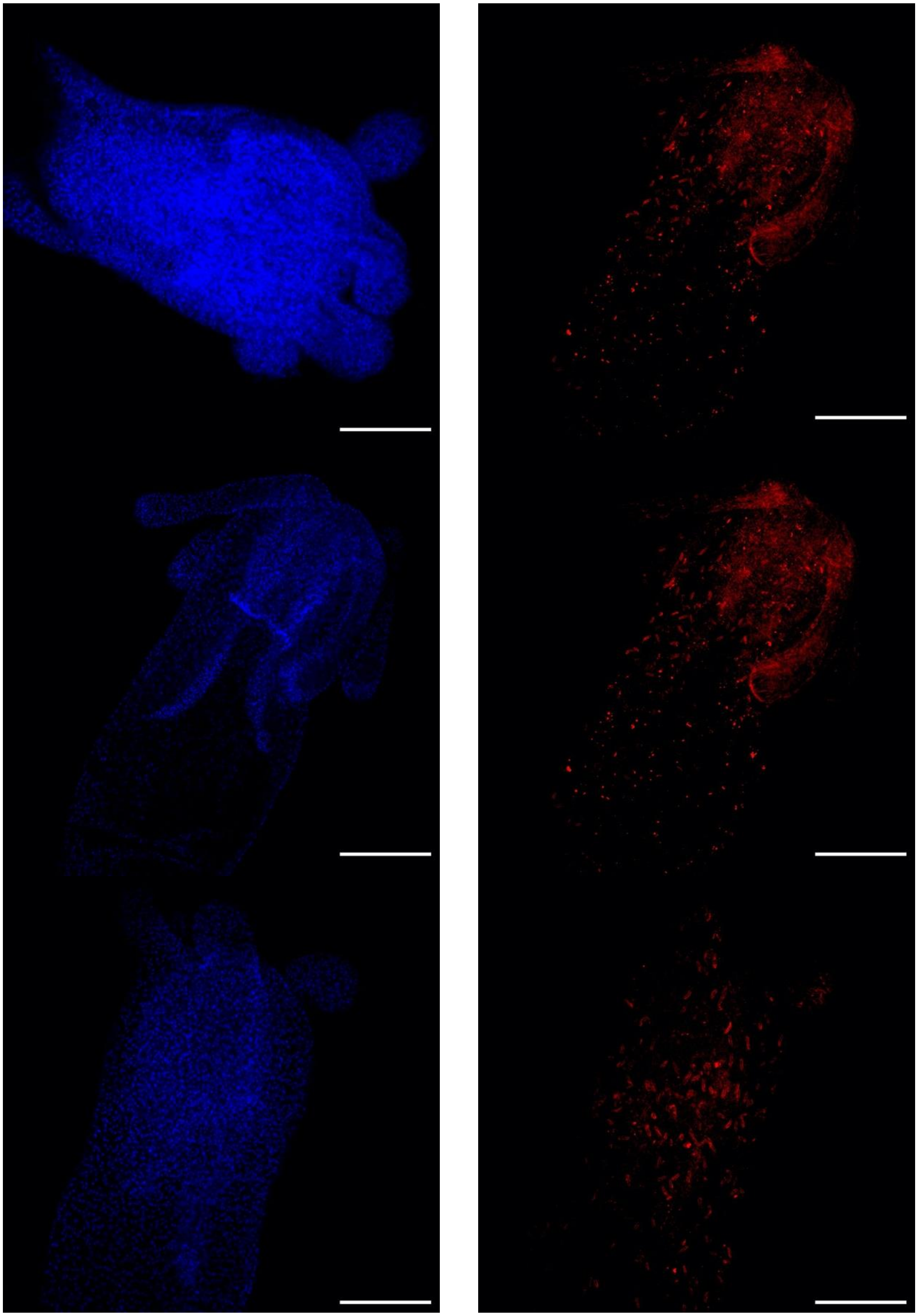

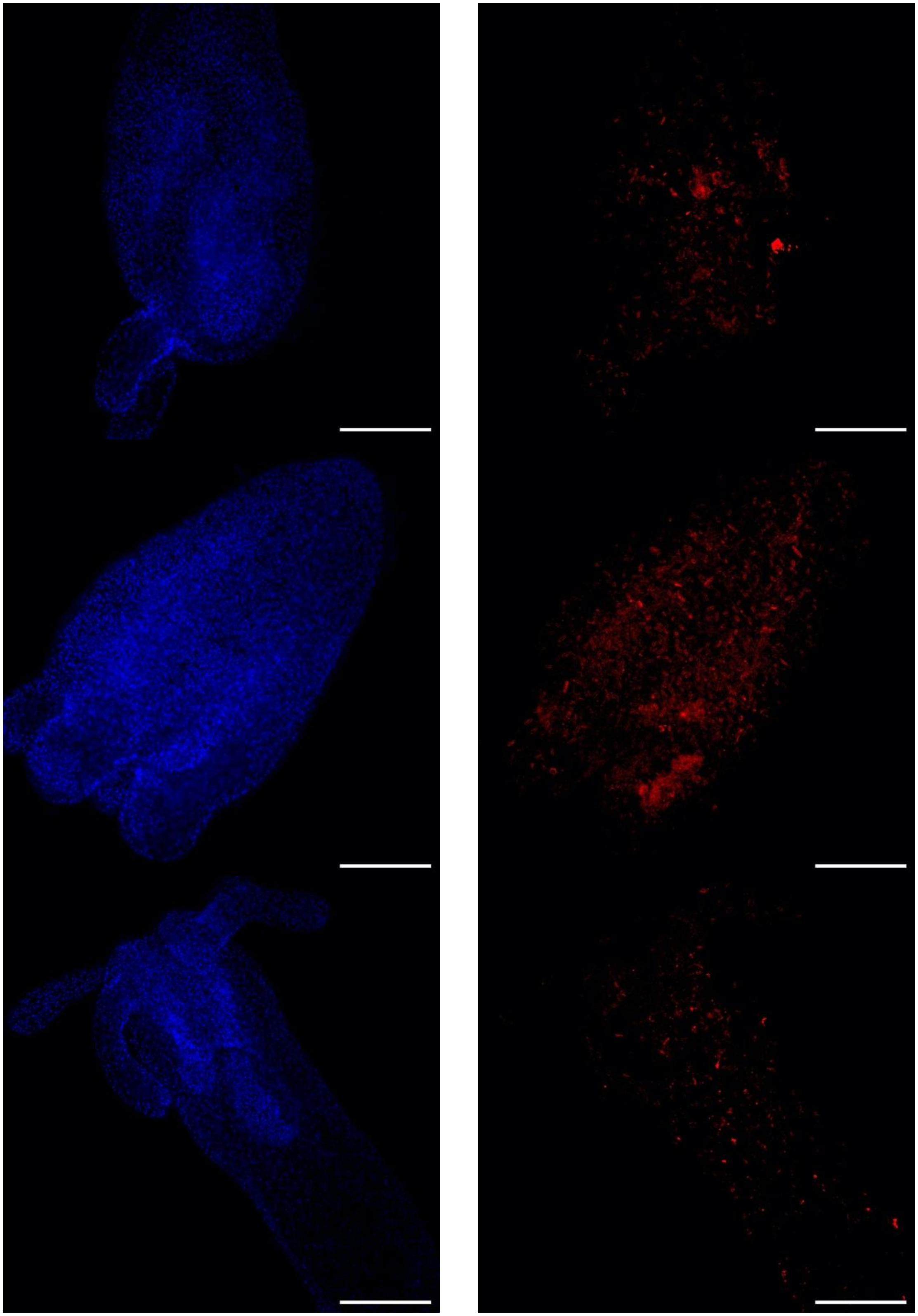

Starved

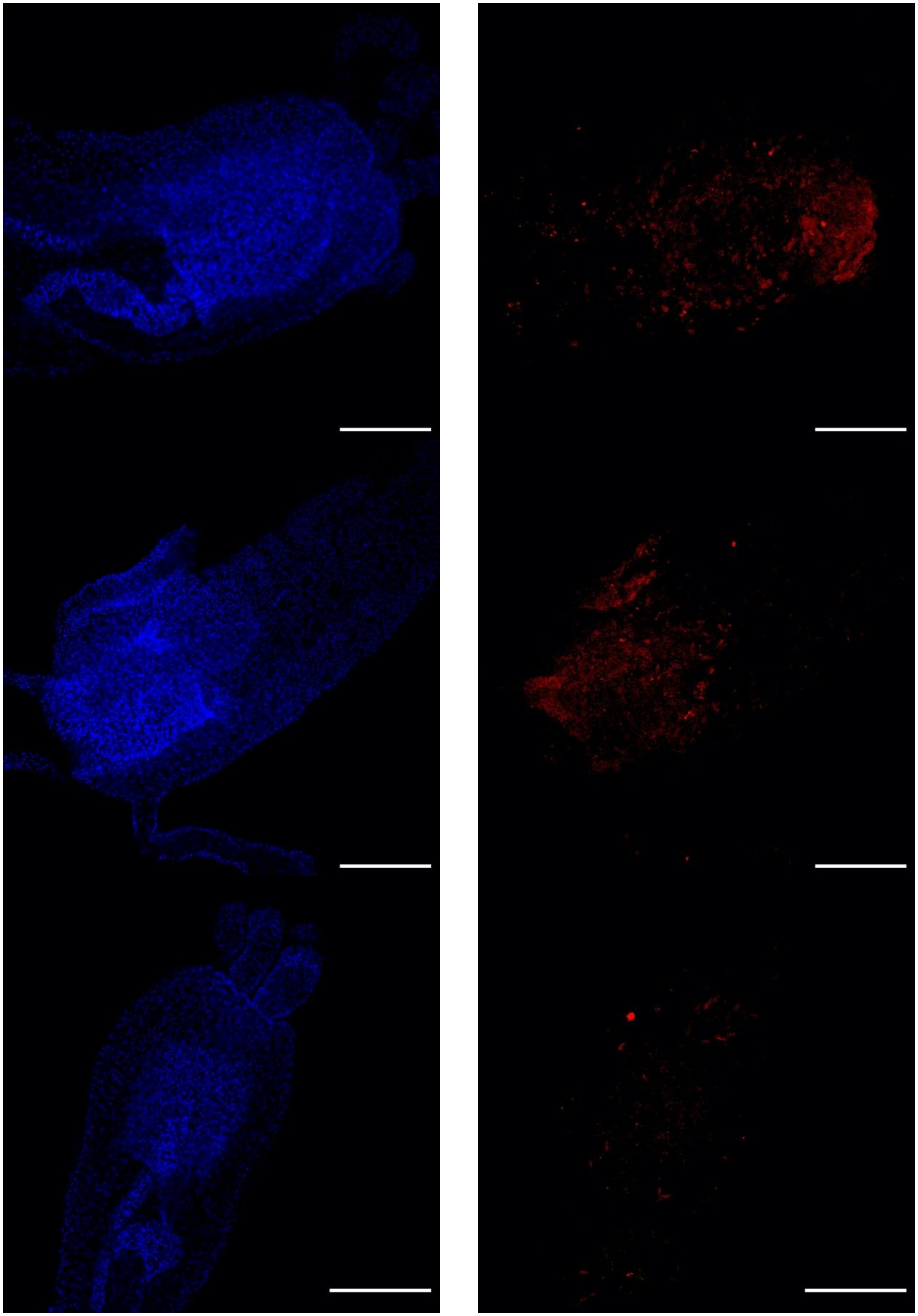

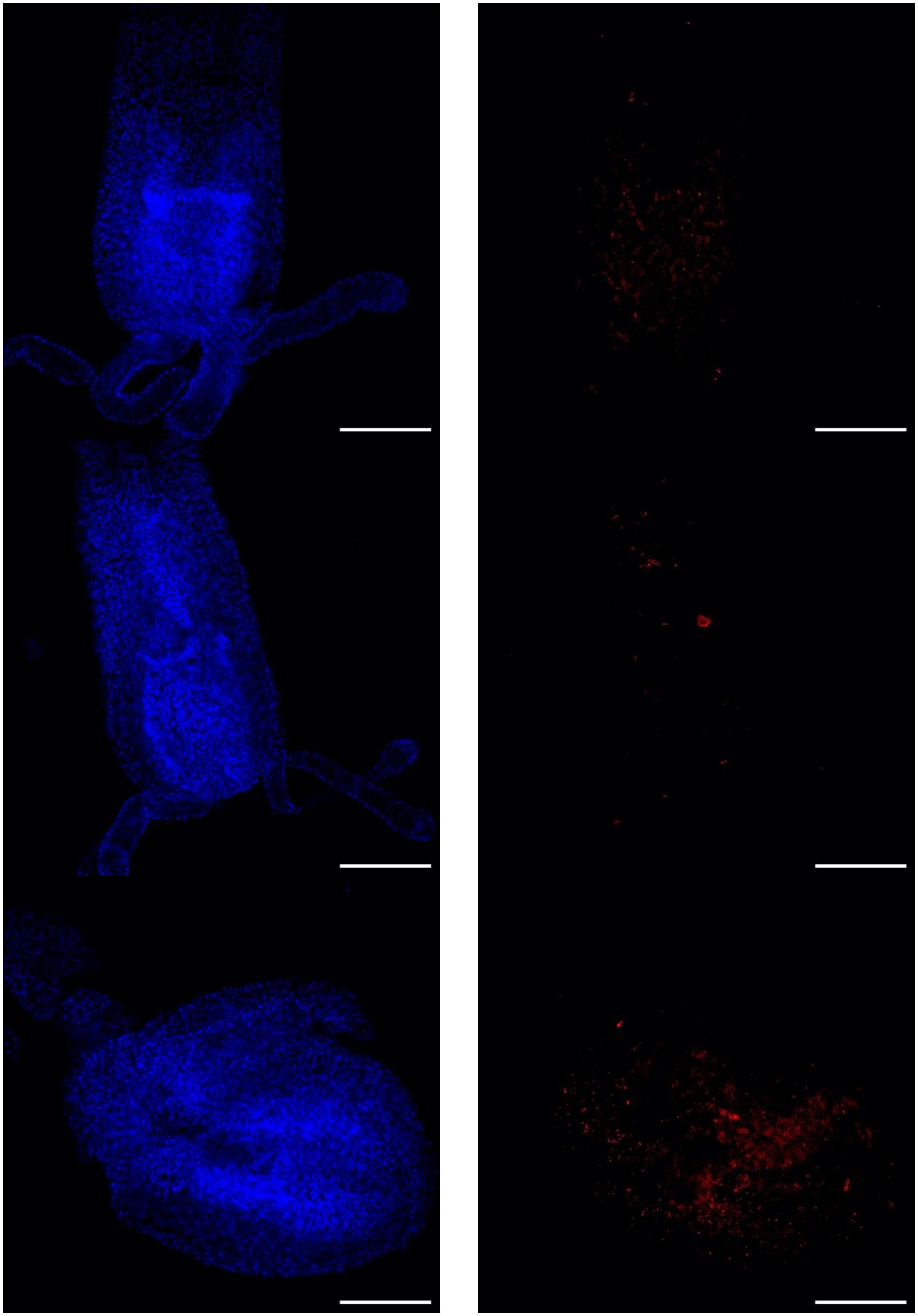

Supplementary Fig. 9 Immunohistochemistry images of fed and starved juvenile anemones used to generate the data for Figs. 3d and 3e in the text.

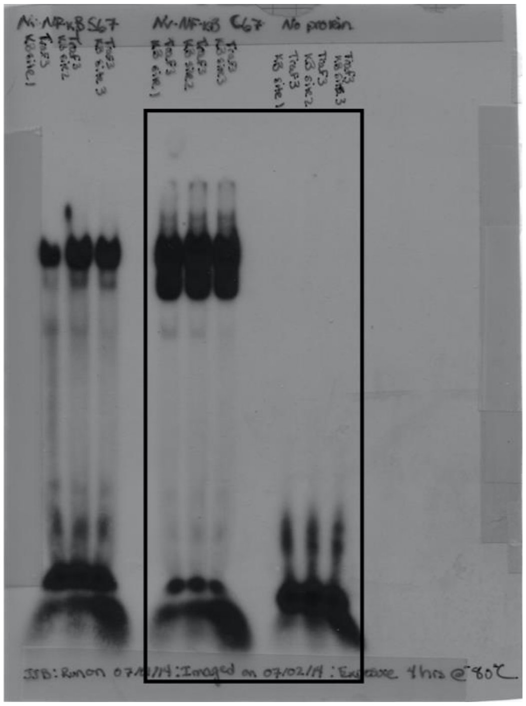

Supplementary Fig. 10 Uncropped version of Fig. 4a. The boxed area was used to make the cropped image for Fig. 4a in the main text.

Supplementary Fig. 4a

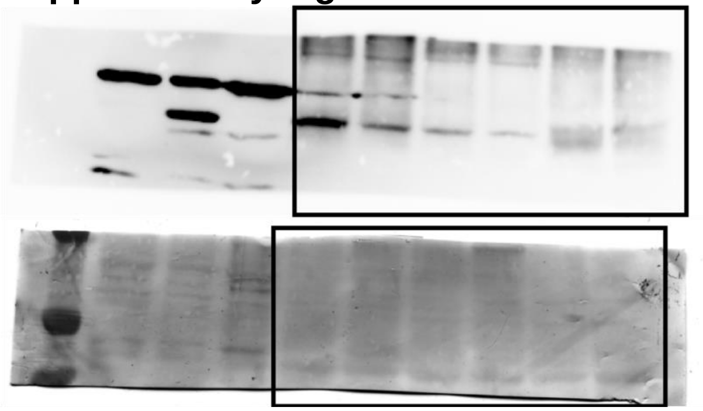

Supplementary Fig. 4b

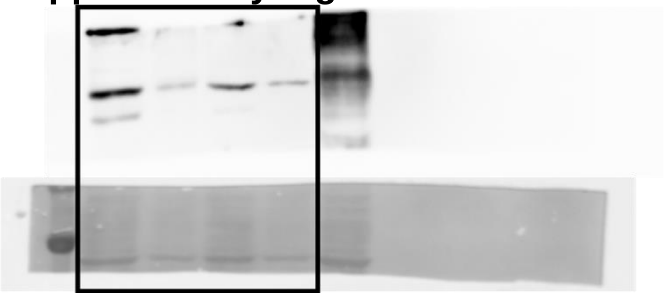

Supplementary Fig. 4c

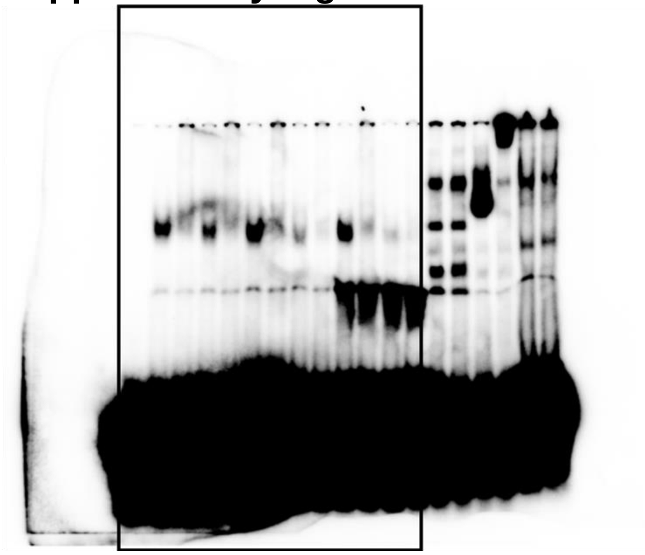

Supplementary Fig. 11 Uncropped version of Supplementary Fig. 4. Boxes indicate areas used to make the cropped images in Supplementary Fig. 4.

Supplementary Table 1. Sample metadata for anemones used in differential gene expression analysis. Table includes sample name, clonal origin, what end (oral/aboral) the anemone regenerated from, feeding regime, file label for sequencing data available on SRA PRJNA837630, and total raw reads for each sample.

Anemone	Clonal parent	Origin end	Feeding regime	File label	Raw reads
1F	1	Oral	Fed	1OF	8,249,165
1S	1	Aboral	Starved	1AS	6,043,016
2F	2	Oral	Fed	2OF	5,891,586
2S	2	Aboral	Starved	2AS	6,095,160
3F	3	Oral	Fed	4OF	9,197,316
3S	3	Aboral	Starved	4AS	7,487,177
4F	4	Aboral	Fed	6AF	5,402,652
4S	4	Oral	Starved	6OS	5,448,963
5F	5	Aboral	Fed	7AF	5,538,452
5S	5	Oral	Starved	7OS	7,403,004
6F	6	Aboral	Fed	8AF	5,215,428
6S	6	Oral	Starved	8OS	8,528,160
7F	7	Aboral	Fed	9AF	7,597,310
7S	7	Oral	Starved	9OS	6,244,255
8F	8	Aboral	Fed	10AF	6,604,076
8S	8	Oral	Starved	10OS	8,152,984

Supplementary Table 2. Anemone survival after immune challenge with *Pseudomonas aeruginosa*. Ten day-old anemones were either fed on a regular schedule for 30 days or starved, and were then infected with 6.8 x 10^8 CFU/ml of *P. aeruginosa* at 28˚C. Survival was monitored daily for 15 days and recorded. These are the original data used to generate the graphs that are presented in Fig. 2 in the main text (Experiment 1 below) and Supplementary Fig. 3 (Experiments 2 and 3).

Experiment 1

	**Surviving anemones**	
**Day**	**Fed**	**Starved**
0	12	12
1	12	12
2	12	12
3	12	12
4	11	9
5	11	7
6	11	3
7	6	2
8	4	2
9	3	0
10	3	0
11	3	0
12	2	0
13	2	0
14	2	0
15	2	0

Experiment 2

	**Surviving anemones**	
**Day**	**Fed**	**Starved**
0	24	24
1	24	21
2	21	3
3	13	1
4	10	0
5	9	0
6	8	0
7	7	0
8	7	0
9	7	0

10	7	0
11	7	0
12	6	0
13	5	0
14	5	0
15	5	0

Experiment 3

	**Surviving anemones**	
**Day**	**Fed**	**Starved**
0	24	24
1	23	20
2	10	0
3	3	0
4	3	0
5	1	0
6	0	0
7	0	0
8	0	0
9	0	0
10	0	0
11	0	0
12	0	0
13	0	0
14	0	0
15	0	0

Supplementary Table 3. Change in NF-κB normalized gene counts between clonal pairs. NF-κB gene counts for Fed and Starved anemones for each clonal pair are presented as the NF-κB transcripts over the total filtered reads per sample. Relative NF-κB mRNA expression was determined by dividing the normalized NF-κB counts for each starved clone by the normalized NF-κB counts of its fed counterpart.

**Clone**	**Fed** **(NF-κB counts/total counts)**	**Starved** **(NF-κB counts/total counts)**	**Relative NF-κB (starved/fed)**
1	26/1828381	12/1224858	0.69
2	11/1133896	3/1152596	0.27
3	24/1982020	8/1494445	0.44
4	8/1194611	13/1243135	1.56
5	25/1254392	11/1612949	0.34
6	14/1204938	23/1784150	1.11
7	20/1670875	10/1456236	0.57
8	20/1410416	5/1601085	0.22
		Average:	0.65

Supplementary Table 4. "Green" module genes with FIMO-predicted Nv-NF-κB-binding sites. The top 50 genes ranked by membership score (kME) (Fig. 4c) where aligned to the *Nv* genome using BLAST, and the 500 bp upstream of the TSS were extracted. Putative Nv-NF-κB-binding sites were searched for using the predicted using a PBM-generated binding motif and FIMO with a p-value cutoff of 7E-05. Gene names are provided where available as annotated in the transcriptome[67]; otherwise transcript names are given. PBM z-score for binding sites from ref.[29] are provided where available; NA, z-score not available, since the site was not on the PBM[29]. Of note, NVE8222 and NVE8223 aligned to the same region of the genome and are likely different transcripts of the same gene.

Gene Name	kB binding site	p-value	PBM z-score
NVE8223	GGGGATTTTC	6.55E-06	13.78
NVE14582	GGGGAAGTCT	5.28E-05	7.93
NVE12208	GGGGACTCTC	5.28E-05	NA
coiled-coil and c2 domain-containing protein 1a	GGGGAAATAT	6.07E-05	13.23
NVE13574	GGGGAAGCCC	6.74E-05	8.58
NVE24104	GGGGAATACC	1.85E-05	10.14
autophagy-related protein 2 homolog a	GGGGAATCCA	3.41E-05	9.54
transmembrane receptor family protein	GGGGAAACTC	5.58E-05	9.96
zinc finger protein 564	GGGGATTCTT	6.56E-05	11.77
NVE8222	GGGGATTTTC	6.55E-06	13.78

Supplementary Table 5. Frequency of FIMO-predicted Nv-NF-κB-binding sites in the upstream regions of randomly selected genes. 200 random gene transcripts were chosen by assigning every transcript in the transcriptome a number and using a random-number generator to select 200 genes. The upstream genomic regions corresponding to these randomly selected transcripts were then searched for possible Nv-NF-κB binding sites. That is, these 200 transcripts were aligned to the *Nv* genome using BLAST, and DNA sequences that were 500 bp upstream of the TSS were extracted. Putative Nv-NF-κB-binding sites were identified using a PBM-generated binding motif and FIMO with a p-value cutoff of 7E-05. 'NA' indicates that no binding site was identified in the region.

Gene name	kB binding site	p-value
NVE12529	GGGGAACTCC	4.59E-05
NVE12828	AGGGGATTCT	5.25E-05
NVE1298	GGGGGAAATT	7.92E-06
NVE13225	GGGGGAATTT	9.09E-07
NVE13394	GGGGGTTTTC	2.88E-05
NVE16790	TGGGAATTTT	5.09E-05
NVE18363	GGGGGAAACA	5.33E-05
NVE19023	GGGGAAATCT	1.22E-05
NVE19807	TGGGAATTTT	5.09E-05
NVE2060	GGGGGTTTCT	4.92E-05
NVE22319	TGGGAAATTT	4.05E-05
NVE22740	GGGGAATTCT	2.56E-06
NVE5198	GGGGACTTTT	3.89E-05
NVE6851	GGGGAATTTG	1.20E-05
NVE8711	GGGGATTTTA	4.00E-05
NVE9216	GGGGATTTTT	2.02E-05
	GGGGATTTCA	2.81E-05
NVE10004	None	NA
NVE10127	None	NA
NVE10482	None	NA
NVE10600	None	NA
NVE1084	None	NA
NVE11081	None	NA
NVE11185	None	NA
NVE1127	None	NA
NVE1154	None	NA
NVE11550	None	NA
NVE11574	None	NA
NVE11717	None	NA
NVE11949	None	NA
NVE1226	None	NA

NVE12370	None	NA
NVE12450	None	NA
NVE12564	None	NA
NVE13001	None	NA
NVE13035	None	NA
NVE13093	None	NA
NVE13199	None	NA
NVE13229	None	NA
NVE1323	None	NA
NVE13386	None	NA
NVE13416	None	NA
NVE13515	None	NA
NVE13539	None	NA
NVE13721	None	NA
NVE13908	None	NA
NVE13910	None	NA
NVE13925	None	NA
NVE14042	None	NA
NVE14129	None	NA
NVE1417	None	NA
NVE14241	None	NA
NVE14863	None	NA
NVE14981	None	NA
NVE15007	None	NA
NVE15011	None	NA
NVE1512	None	NA
NVE15240	None	NA
NVE15386	None	NA
NVE15518	None	NA
NVE15893	None	NA
NVE1594	None	NA
NVE15959	None	NA
NVE16291	None	NA
NVE16509	None	NA
NVE16538	None	NA
NVE16598	None	NA
NVE17004	None	NA
NVE17173	None	NA
NVE17260	None	NA
NVE17333	None	NA

NVE17511	None	NA
NVE17537	None	NA
NVE17548	None	NA
NVE17692	None	NA
NVE17780	None	NA
NVE17820	None	NA
NVE17902	None	NA
NVE18014	None	NA
NVE18056	None	NA
NVE18376	None	NA
NVE18506	None	NA
NVE18534	None	NA
NVE18541	None	NA
NVE18893	None	NA
NVE19164	None	NA
NVE19193	None	NA
NVE1921	None	NA
NVE19360	None	NA
NVE1940	None	NA
NVE19426	None	NA
NVE19502	None	NA
NVE19569	None	NA
NVE19831	None	NA
NVE20005	None	NA
NVE20081	None	NA
NVE20249	None	NA
NVE2031	None	NA
NVE20447	None	NA
NVE2057	None	NA
NVE20619	None	NA
NVE20923	None	NA
NVE21172	None	NA
NVE21182	None	NA
NVE21356	None	NA
NVE21440	None	NA
NVE21466	None	NA
NVE21541	None	NA
NVE219	None	NA
NVE21919	None	NA
NVE21924	None	NA

NVE21951	None	NA
NVE22754	None	NA
NVE23034	None	NA
NVE23492	None	NA
NVE2379	None	NA
NVE23938	None	NA
NVE23998	None	NA
NVE24048	None	NA
NVE2435	None	NA
NVE24362	None	NA
NVE24451	None	NA
NVE24523	None	NA
NVE24600	None	NA
NVE24654	None	NA
NVE24678	None	NA
NVE24702	None	NA
NVE24703	None	NA
NVE24897	None	NA
NVE25075	None	NA
NVE25108	None	NA
NVE25316	None	NA
NVE25336	None	NA
NVE25440	None	NA
NVE25540	None	NA
NVE25563	None	NA
NVE25674	None	NA
NVE25842	None	NA
NVE25886	None	NA
NVE25908	None	NA
NVE25910	None	NA
NVE2631	None	NA
NVE2639	None	NA
NVE2645	None	NA
NVE2764	None	NA
NVE3013	None	NA
NVE3037	None	NA
NVE3610	None	NA
NVE362	None	NA
NVE3879	None	NA
NVE4108	None	NA

NVE4288	None	NA
NVE4335	None	NA
NVE4424	None	NA
NVE4479	None	NA
NVE4556	None	NA
NVE4564	None	NA
NVE4585	None	NA
NVE473	None	NA
NVE4793	None	NA
NVE4881	None	NA
NVE4986	None	NA
NVE5127	None	NA
NVE5240	None	NA
NVE5560	None	NA
NVE5736	None	NA
NVE5821	None	NA
NVE5835	None	NA
NVE5880	None	NA
NVE6024	None	NA
NVE6314	None	NA
NVE6321	None	NA
NVE640	None	NA
NVE655	None	NA
NVE6608	None	NA
NVE681	None	NA
NVE6839	None	NA
NVE7034	None	NA
NVE709	None	NA
NVE7105	None	NA
NVE7189	None	NA
NVE7242	None	NA
NVE7433	None	NA
NVE7621	None	NA
NVE8029	None	NA
NVE8067	None	NA
NVE8347	None	NA
NVE8571	None	NA
NVE882	None	NA
NVE9283	None	NA
NVE9294	None	NA

NVE9354	None	NA
NVE963	None	NA
NVE9829	None	NA
NVE9896	None	NA
NVE11187	None	NA
NVE12533	None	NA
NVE16772	None	NA
NVE17697	None	NA
NVE24946	None	NA
NVE4590	None	NA

Supplementary Table 6. Cell counts of NF-κB-positive cells in juvenile *Nv* for both feeding treatments. Each row indicates NF-κB-positive cells for an individual anemone per condition. These data were used to generate the graph in Fig. 3e in the main text.

**Fed**	**Starved**
213	111
91	146
169	49
169	91
411	19
140	62

Supplementary Table 7. Luciferase assay values as measured in Relative Light Units (RLU). These data were used to make the graph in Fig. 4b in the main text.

	RLU	B-gal activity	Normalized RLU	Average Normalized RLU	Relative Average Normalized RLU
Traf3Luc No (-) NFkB	307826	0.872	352963.3		
	265020	0.614	4315603	392063.7	1.0
	143784	0.367	391667.6		
Traf3Luc plus (+) NFkB	509545	0.404	1261146		
	258776	0.204	1268303.9	1257831.0	3.2
	634504	0.510	1244043.1		
Traf3mutLuc No (-) NFkB	215697	0.591	364898.5		
	415715	0.824	503236.1	455122.4	1.0
	241697	0.486	497232.5		
Traf3mutLuc plus (+) NFkB	100567	0.280	359017.9		
	181699	0.388	468188.1	410028.8	0.9
	119051	0.295	364898.5		

Dataset S1 (separate file). List of significant DEGs (*FDR adjusted p-value* < 0.1) in starved anemones relative to fed controls. The table in Dataset S1 includes output data generated by *DESeq2*.
