# Supplementary material for: Starvation decreases immunity and immune regulatory factor NF-κB in the starlet sea anemone Nematostella vectensis
Source: Commun Biol. 2023 Jul 7;6:698. doi: 10.1038/s42003-023-05084-7 (PMC10329013; doi:10.1038/s42003-023-05084-7)
Supplement: Supplementary file 2 — Description of Additional Supplementary Files [file 42003_2023_5084_MOESM2_ESM.pdf]

Description of Additional Supplementary Files

File name: Supplementary Data 1

Description: A full list of the differentially expressed genes.
