# Supplementary material for: Starvation decreases immunity and immune regulatory factor NF-κB in the starlet sea anemone Nematostella vectensis
Source: Commun Biol. 2023 Jul 7;6:698. doi: 10.1038/s42003-023-05084-7 (PMC10329013; doi:10.1038/s42003-023-05084-7)
Supplement: Supplementary file 4 — Reporting Summary [file 42003_2023_5084_MOESM4_ESM.pdf]

nature portfolio

Corresponding author(s): Thomas Gilmore

Last updated by author(s): June 17, 2023

Reporting Summary

Nature Portfolio wishes to improve the reproducibility of the work that we publish. This form provides structure for consistency and transparency in reporting. For further information on Nature Portfolio policies, see our Editorial Policies and the Editorial Policy Checklist.

Statistics

For all statistical analyses, confirm that the following items are present in the figure legend, table legend, main text, or Methods section.

n/a	Confirmed	
☐	☒	The exact sample size (*n*) for each experimental group/condition, given as a discrete number and unit of measurement
☐	☒	A statement on whether measurements were taken from distinct samples or whether the same sample was measured repeatedly
☐	☒	The statistical test(s) used AND whether they are one- or two-sided<br>*Only common tests should be described solely by name; describe more complex techniques in the Methods section.*
☐	☒	A description of all covariates tested
☐	☒	A description of any assumptions or corrections, such as tests of normality and adjustment for multiple comparisons
☐	☒	A full description of the statistical parameters including central tendency (e.g. means) or other basic estimates (e.g. regression coefficient) AND variation (e.g. standard deviation) or associated estimates of uncertainty (e.g. confidence intervals)
☐	☒	For null hypothesis testing, the test statistic (e.g. *F*, *t*, *r*) with confidence intervals, effect sizes, degrees of freedom and *P* value noted<br>*Give P values as exact values whenever suitable.*
☒	☐	For Bayesian analysis, information on the choice of priors and Markov chain Monte Carlo settings
☐	☒	For hierarchical and complex designs, identification of the appropriate level for tests and full reporting of outcomes
☒	☐	Estimates of effect sizes (e.g. Cohen's *d*, Pearson's *r*), indicating how they were calculated

Our web collection on statistics for biologists contains articles on many of the points above.

Software and code

Policy information about availability of computer code

Data collection	Protocol for library preparation is available at https://github.com/z0on/tag-based_RNAseq.
Data analysis	Scripts for TagSeq analysis are available at https://github.com/z0on/tag-based_RNAseq. Protocol for Gene Ontology analysis is available at https://github.com/z0on/GO_MWU. Scripts for all other analysis used in this publication are available at https://github.com/joshuaguirre29/Nematostella_nutrition_and_starvation.

For manuscripts utilizing custom algorithms or software that are central to the research but not yet described in published literature, software must be made available to editors and reviewers. We strongly encourage code deposition in a community repository (e.g. GitHub). See the Nature Portfolio guidelines for submitting code & software for further information.

Data

Policy information about availability of data

All manuscripts must include a data availability statement. This statement should provide the following information, where applicable:

- Accession codes, unique identifiers, or web links for publicly available datasets
- A description of any restrictions on data availability
- For clinical datasets or third party data, please ensure that the statement adheres to our policy

Nematostella vectensis transcriptome can be found at https://figshare.com/articles/dataset/Nematostella_vectensis_transcriptome_and_gene_models_v2_0/807696. High-resolution images of Supplementary Figs. 1 and 7 are available at https://

github.com/joshuaguirre29/Nematostella_nutrition_and_starvation. Raw reads have been submitted to SRA under PRJNA837630. Additional data relating to the study are available from the corresponding author upon reasonable request.

Research involving human participants, their data, or biological material

Policy information about studies with human participants or human data. See also policy information about sex, gender (identity/presentation), and sexual orientation and race, ethnicity and racism.

Reporting on sex and gender	*Use the terms sex (biological attribute) and gender (shaped by social and cultural circumstances) carefully in order to avoid confusing both terms. Indicate if findings apply to only one sex or gender; describe whether sex and gender were considered in study design; whether sex and/or gender was determined based on self-reporting or assigned and methods used. Provide in the source data disaggregated sex and gender data, where this information has been collected, and if consent has been obtained for sharing of individual-level data; provide overall numbers in this Reporting Summary. Please state if this information has not been collected. Report sex- and gender-based analyses where performed, justify reasons for lack of sex- and gender-based analysis.*
Reporting on race, ethnicity, or other socially relevant groupings	*Please specify the socially constructed or socially relevant categorization variable(s) used in your manuscript and explain why they were used. Please note that such variables should not be used as proxies for other socially constructed/relevant variables (for example, race or ethnicity should not be used as a proxy for socioeconomic status). Provide clear definitions of the relevant terms used, how they were provided (by the participants/respondents, the researchers, or third parties), and the method(s) used to classify people into the different categories (e.g. self-report, census or administrative data, social media data, etc.) Please provide details about how you controlled for confounding variables in your analyses.*
Population characteristics	*Describe the covariate-relevant population characteristics of the human research participants (e.g. age, genotypic information, past and current diagnosis and treatment categories). If you filled out the behavioural & social sciences study design questions and have nothing to add here, write "See above."*
Recruitment	*Describe how participants were recruited. Outline any potential self-selection bias or other biases that may be present and how these are likely to impact results.*
Ethics oversight	*Identify the organization(s) that approved the study protocol.*

Note that full information on the approval of the study protocol must also be provided in the manuscript.

Field-specific reporting

Please select the one below that is the best fit for your research. If you are not sure, read the appropriate sections before making your selection.

☒ Life sciences ☐ Behavioural & social sciences ☐ Ecological, evolutionary & environmental sciences

For a reference copy of the document with all sections, see nature.com/documents/nr-reporting-summary-flat.pdf

Life sciences study design

All studies must disclose on these points even when the disclosure is negative.

Sample size	Sample size for TagSeq library preparation was chosen to give statistically significant data with the possibility that outliers could be detected, however none was identified in this analysis. Samples sizes for experiments such as immunofluorescence and reporter gene assays were chosen to give statistically significant data according to standard experiments in the field. For example, with immunofluorescence, we analyzed 6 anemones per feeding condition, and for reported gene assays, experiments were from at least three biologically independent experiments performed with triplicate samples.
Data exclusions	As described in the Methods, sequences that were <20 bp with <90% of bases having quality cut-off scores >20 were trimmed from TagSeq analysis, as commonly done for this type of analysis.
Replication	Experiments were replicated and appropriate measures were taken to verify reproducibility.
Randomization	Randomization was not needed as the use of clonal pairs in both fed and starved conditions is an important feature of our study. In addition, we used SNP analysis to confirm that biological pairs of anemones were valid.
Blinding	Blinding is not relevant to our study, as all experiments involved direct comparision of data between samples in the given group, e.g., for control vectors in Western blots, EMSAs, reporter gene assays, immunoprecipitations, and indirect immunofluorescence.

Reporting for specific materials, systems and methods

We require information from authors about some types of materials, experimental systems and methods used in many studies. Here, indicate whether each material, system or method listed is relevant to your study. If you are not sure if a list item applies to your research, read the appropriate section before selecting a response.

Materials & experimental systems

n/a	Involved in the study	
☐	☒	Antibodies
☐	☒	Eukaryotic cell lines
☒	☐	Palaeontology and archaeology
☐	☒	Animals and other organisms
☒	☐	Clinical data
☒	☐	Dual use research of concern
☒	☐	Plants

Methods

n/a	Involved in the study	
☒	☐	ChIP-seq
☒	☐	Flow cytometry
☒	☐	MRI-based neuroimaging

Antibodies

Antibodies used	Anti-Nv-NF-kB antiserum; Horseradish peroxidase-conjugated anti-rabbit antiserum (Cell Signaling, Cat#7074); Texas-red-conjugated anti-rabbit antiserum (Invitrogen, Cat#T-2767)
Validation	Antibodies were verified by using negative controls in experiments (e.g., vector alone) and the identification of specific bands in Western blots of the predicted sizes.

Eukaryotic cell lines

Policy information about cell lines and Sex and Gender in Research

Cell line source(s)	HEK293 cells (ATCC)
Authentication	Cell lines were authenticated by suppliers
Mycoplasma contamination	Cell lines were not tested for mycoplasma contamination. However, the experiments performed herein did not characterize specific properties of the cells, rather they used the cells as vehicles for overexpressing exogenous proteins.
Commonly misidentified lines (See ICLAC register)	None

Animals and other research organisms

Policy information about studies involving animals; ARRIVE guidelines recommended for reporting animal research, and Sex and Gender in Research

Laboratory animals	Nematostella vectensis, Maryland population, adults and juveniles.
Wild animals	Study did not involve wild animals.
Reporting on sex	Anemone sex was not determined prior to experimentation. The use of clonal pairs as described in the manuscript, controls for both sexes. Sex in juveniles anemones was not relevant to the pooled studies reporter herein.
Field-collected samples	Study did not involve samples collected from the field.
Ethics oversight	No ethical oversight was needed since the animals in question are 'lower' invertebrates, cnidarians.

Note that full information on the approval of the study protocol must also be provided in the manuscript.
